# Art2 mediates selective endocytosis of methionine transporters during adaptation to sphingolipid depletion

**DOI:** 10.1242/jcs.260675

**Published:** 2023-07-25

**Authors:** Nathaniel L. Hepowit, Bradley Moon, Adam C. Ebert, Robert C. Dickson, Jason A. MacGurn

**Affiliations:** ^1^ Department of Cell and Developmental Biology, Vanderbilt University, Nashville, TN 37240, USA; ^2^ Department of Molecular and Cellular Biochemistry, University of Kentucky, Lexington, KY 40536, USA

**Keywords:** Sphingolipid metabolism, Endocytosis, Endocytic adaptors, Amino acid transporters, Methionine transport, Glucose transport, α-arrestins, Ubiquitin, Myriocin

## Abstract

Accumulating evidence in several model organisms indicates that reduced sphingolipid biosynthesis promotes longevity, although underlying mechanisms remain unclear. In yeast, sphingolipid depletion induces a state resembling amino acid restriction, which we hypothesized might be due to altered stability of amino acid transporters at the plasma membrane. To test this, we measured surface abundance for a diverse panel of membrane proteins in the presence of myriocin, a sphingolipid biosynthesis inhibitor, in *Saccharomyces cerevisiae*. Unexpectedly, we found that surface levels of most proteins examined were either unaffected or increased during myriocin treatment, consistent with an observed decrease in bulk endocytosis. In contrast, sphingolipid depletion triggered selective endocytosis of the methionine transporter Mup1. Unlike methionine-induced Mup1 endocytosis, myriocin triggered Mup1 endocytosis that required the Rsp5 adaptor Art2, C-terminal lysine residues of Mup1 and the formation of K63-linked ubiquitin polymers. These findings reveal cellular adaptation to sphingolipid depletion by ubiquitin-mediated remodeling of nutrient transporter composition at the cell surface.

## Teaser

**Summary:** Sphingolipid depletion triggers plasma membrane remodeling by altering endocytosis of amino acid transporters.

## INTRODUCTION

Sphingolipids (SLs) are a diverse class of lipids that serve as a structural component of eukaryotic membranes but also have important regulatory functions related to cell signaling. The first steps of SL biosynthesis occur in the endoplasmic reticulum (ER) and result in the production of ceramide, which is then transported to the Golgi complex for further modification into complex SLs. These complex SLs are then transported to different membranes throughout the cell where they serve a multitude of functions (Körner and Fröhlich, 2022). For example, sphingomyelin regulates sorting of specific secretory cargo in the trans-Golgi network of mammalian cells (Deng et al., 2018; Sundberg et al., 2019). In the plasma membrane (PM) of mammalian cells, sphingosine-1-phosphate is generated and can be secreted to act as a signaling molecule that mediates complex processes, including vascular development and coordination of immune responses (Cartier and Hla, 2019). In yeast, SLs at the PM regulate the activation of TORC2 (Berchtold et al., 2012). Indeed, the variety of regulatory functions served by SLs is underscored by their important role in processes that range from memory and cognition (Kalinichenko et al., 2022) to the progression of cancer (Bataller et al., 2021; Janneh and Ogretmen, 2022).

Myriocin (Myr) is a potent inhibitor of serine palmitoyltransferase (SPT), which catalyzes the first step of SL biosynthesis and increases lifespan in a variety of model organisms (Huang et al., 2014). There is a growing body of data showing that Myr treatment reduces the severity of age-related diseases in mice and rats, including atherosclerosis and cardiac impairment (Glaros et al., 2010; Jiang et al., 2011; Summers et al., 2019; Yu et al., 2020), factors for metabolic syndrome, obesity, diabetes and cancer (Aburasayn et al., 2016; Appriou et al., 2019; Selwan et al., 2016; Woo et al., 2019; Yaguchi et al., 2017), and amyloid-β and tau hyperphosphorylation in Alzheimer's disease (Geekiyanage et al., 2013) and other neurodegenerative diseases (Lin et al., 2019; Petit et al., 2020). Despite its therapeutic potential, it remains unclear how dampening SL biosynthesis confers these health benefits to enhance longevity.

Perturbations that alter SL homeostasis have complex effects on cellular processes. By characterizing how *Saccharomyces cerevisiae* yeast cells respond and adapt to Myr treatment, we have worked to understand how SL depletion promotes longevity. Recently, we reported that Myr-treated yeast cells experience a state resembling amino acid restriction, which is associated with decreased uptake of amino acids from the medium into the cell (Hepowit et al., 2021). We also reported that Myr triggered the endocytic clearance of the high-affinity methionine transporter Mup1 (Hepowit et al., 2021). Based on these prior results, we hypothesized that SL depletion might trigger broad endocytic clearance of various nutrient transporters. Here, we tested this hypothesis by measuring the surface abundance of a diverse panel of PM proteins, including amino acid transporters (AATs), hexose transporters, proton pumps and signaling receptors. We report that, for most proteins examined, SL depletion either increased the PM abundance or had no apparent effect on subcellular distribution. Consistent with these observations, we found that Myr inhibited bulk endocytosis while simultaneously triggering selective endocytic clearance of Mup1. We also address the mechanism of Myr-mediated Mup1 endocytosis, which is mechanistically distinct from that of methionine-induced Mup1 endocytosis. These studies are crucial to understanding how Myr treatment leads to a state of amino acid restriction, and they provide new insights into how the PM is remodeled in response to SL depletion.

## RESULTS

### Myr triggers remodeling of AAT composition at the PM

**Fig. 1.**
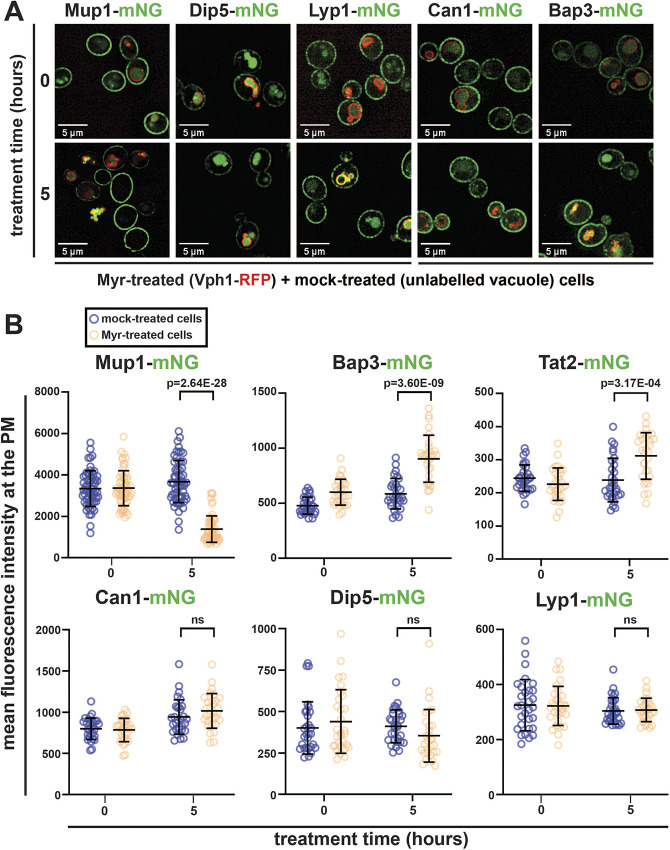
**Myr treatment selectively decreases plasma membrane abundance of the primary methionine permease Mup1.** (A) Yeast cells expressing the indicated amino acid transporters (AATs) with a C-terminal mNG tag (green) were visualized under a fluorescence microscope after treatment with Myr (400 ng ml^−1^) or mock solution (95% ethanol). Myr-treated cells expressed MARS- or mCherry-tagged Vph1 (a vacuolar marker, red; indicated as Vph1–RFP) and mock-treated cells have unlabeled vacuoles. Note that Myr-treated cells expressing Mup1–mNG, Dip5–mNG and Lyp1–mNG also expressed Vph1–MARS, whereas Myr-treated cells expressing Can1–mNG and Bap3–mNG also expressed Vph1–mCherry. Images are representative of triplicate experiments. (B) Quantification of the mean fluorescence intensity of the indicated AAT–mNG at the PM, measured using Fiji (*n*=30–60 cells from triplicate experiments). Bars show the mean±s.d. ns, not significant (one-tailed paired *t*-test).

Our previous finding that Myr treatment generally decreases the intracellular pool of amino acids as well as the rate of amino acid uptake (Hepowit et al., 2021) raised the possibility that gradual SL depletion alters the composition of AATs at the PM. To explore this possibility, we examined cells harboring chromosomal mNeonGreen (mNG) fusions to a panel of AAT genes (*MUP1*, *CAN1*, *DIP5*, *LYP1*, *BAP2*, *BAP3*, *GNP1*, *HIP1*, *GAP1* and *TAT2*) in wildtype (WT) (SEY6210) and Vph1–RFP (a protein localized to the limiting membrane of the vacuole)-expressing background strains. Cells at mid-log phase were treated with Myr (400 ng ml^−1^) or mock treated (solvent) for 5 h and mixed just prior to visualization by fluorescence microscopy. Vph1–RFP expression was used to identify cells that had been treated with Myr, whereas mock treatment was performed on cells lacking any RFP expression. Quantification of images was performed by measuring mean fluorescence intensity at the PM of individual cells. This analysis revealed that Myr treatment did not significantly alter PM levels of Can1, Dip5, Lyp1, Bap2, Gnp1 or Hip1 (Fig. 1A,B; Fig. S1A–C). In the conditions of this experiment, Gap1 was predominantly localized to vacuoles in both mock-treated and Myr-treated cells (Fig. S1A), and thus localization to the PM could not be quantified. In contrast, increased levels of Bap3 and Tat2 at the PM were observed in Myr-treated cells (Fig. 1A,B). Consistent with previously reported results, Myr-treated cells exhibited decreased Mup1–mNG at the PM and increased Mup1–mNG flux into the vacuole (Fig. 1A,B). Taken together, these results indicate that SL depletion induces a selective endocytic clearance of the methionine transporter Mup1.

### Yeast cells respond to Myr by increasing abundance of specific glucose transporters

**Fig. 2.**
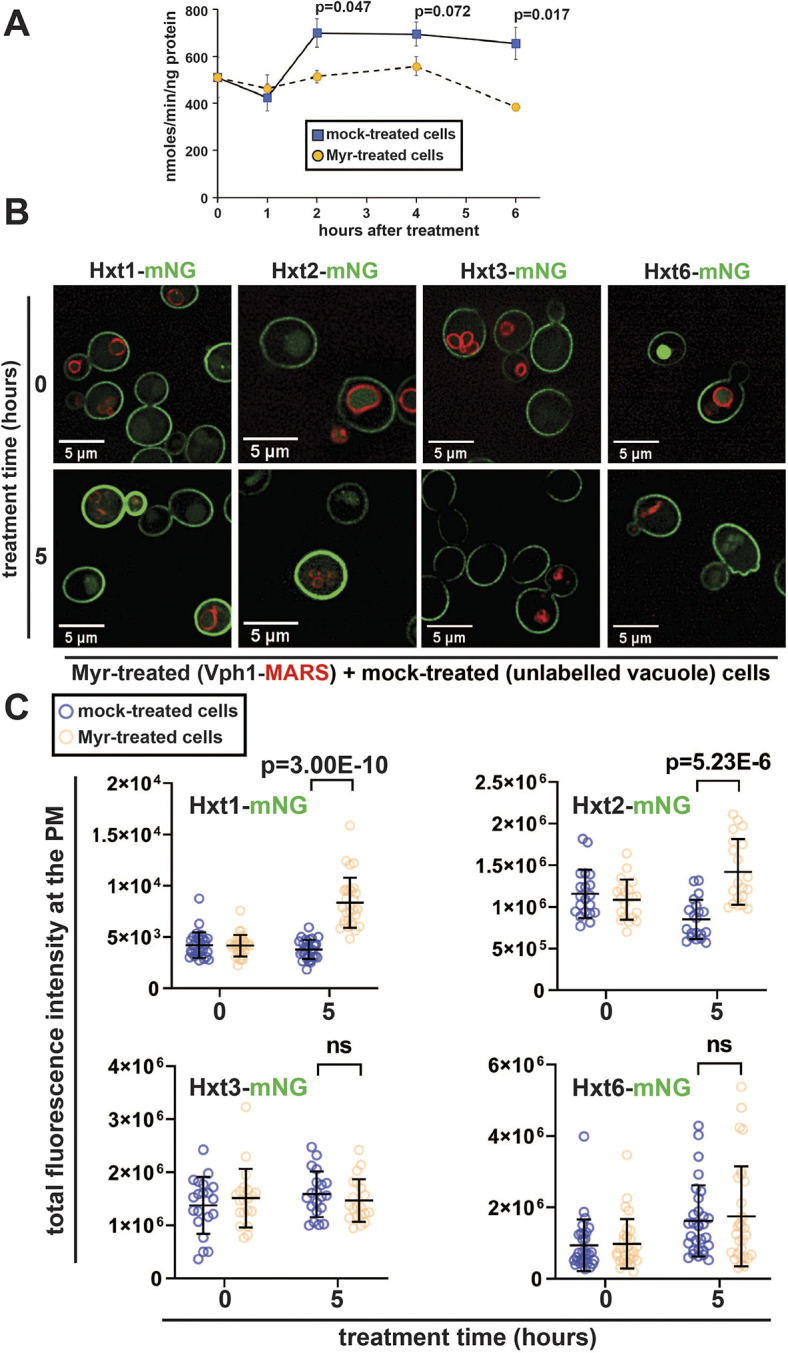
**Myr decreases the cellular uptake of glucose despite increased PM abundance of the glucose transporters Hxt1 and Hxt2.** (A) Time-course measurements of ^3^H-glucose uptake in mock-treated versus Myr-treated yeast cells (*n*=3). Bars show the mean±s.d. *P*-values were calculated using a one-tailed paired *t*-test. (B) Yeast cells expressing the indicated glucose transporters with a C-terminal mNG tag (green) were visualized under a fluorescence microscope after treatment with Myr (400 ng ml^−1^) or mock solution (95% ethanol). Myr-treated cells expressed MARS-tagged Vph1 (a vacuolar marker, red) and mock-treated cells have unlabeled vacuoles. Images are representative of triplicate experiments. (C) Quantification of the total fluorescence intensity of select glucose transporters (C-terminally tagged with mNG) at the PM, measured using Fiji (*n*=30–60 cells from triplicate experiments). Bars show the mean±s.d. ns, not significant (one-tailed paired *t*-test).

Given that yeast cells respond to Myr by altering the PM composition of AATs, we considered the possibility that other nutrient transporters might also be affected. Specifically, given the relationship between glucose metabolism and aging, we hypothesized that Myr treatment might alter the composition or activity of glucose transporters. To examine the effect of Myr on glucose transport, yeast cells were incubated with radiolabeled ^3^H-glucose and uptake was measured with or without Myr treatment. Notably, glucose uptake capacity was reduced in cells treated with Myr for at least 2 h (Fig. 2A). To determine whether Myr treatment affects the abundance of glucose transporters at the PM, we examined cells harboring chromosomal mNG fusions to a panel of four hexose transporter genes (*HXT1*, *HXT2*, *HXT3* and *HXT6*) in WT (SEY6210) and vacuolar Vph1–MARS-expressing background strains. Cells were grown to mid-log phase, treated with Myr or mock treated for 5 h, and mixed just prior to visualization by fluorescence microscopy (as described in Fig. 1). We found that Myr treatment had no effect on the PM abundance of Hxt3 or Hxt6, whereas the low-affinity glucose transporter Hxt1 and the high-affinity glucose transporter Hxt2 exhibited increased abundance at the PM (Fig. 2B,C; Fig. S2A,B). Thus, Myr-treated cells selectively increase PM abundance of specific hexose transporters while experiencing decreased capacity for glucose uptake. These results are unexpected and suggest impaired or suppressed activity of glucose transporters in SL-depleted cells.

We expanded our analysis to include other categories of integral membrane proteins at the PM. Myr-treatment resulted in a slight (but statistically insignificant) decrease in the PM abundance of Pma1, a P2-type ATPase that pumps protons out of the cell (Fig. S2C,D). Myr treatment induced a correspondingly slight (but statistically significant) increase in the PM abundance of Pma2 (Fig. S2C,D), a paralog of Pma1. Although these changes were subtle, Myr treatment induced more substantial increases in the abundance of other proteins at the PM. For example, the pheromone receptor Ste2 exhibited largely vacuolar localization in mock-treated cells but localized to the PM in Myr-treated cells (Fig. S2C,D), suggesting that Myr treatment interferes with constitutive endocytic trafficking of Ste2. Similar results were observed for the stress-sensing signal transducer Wsc1 (also known as Slg1) (Fig. S2C,D). Interestingly, Myr treatment also increased the PM abundance of the ABC family multidrug transporter Pdr5 (Fig. S2C,D). A summary of the Myr treatment response of all integral PM proteins examined is provided in Table 1.

**Table 1.**
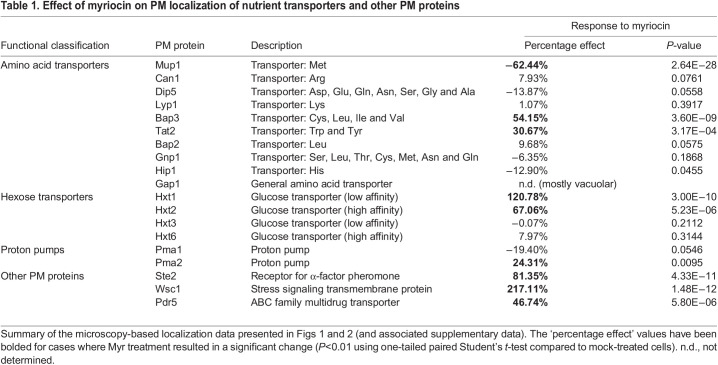
Effect of myriocin on PM localization of nutrient transporters and other PM proteins

Given that several PM proteins exhibited increased surface levels in response to Myr treatment (Table 1), we considered the possibility that cells respond to SL depletion by increasing expression of certain PM proteins. To test this, we examined published data reporting the transcriptional response to Myr treatment (Hepowit et al., 2023) and found that many PM proteins that accumulated at the surface during Myr treatment (e.g. Bap3, Tat2, Hxt1, Ste2 and Wsc1) did not exhibit a corresponding upregulation of transcription (Fig. S3). Two noted exceptions were Hxt2 and Pdr5, which exhibited an upregulation of transcription that corresponded to increased PM levels (Fig. S3). Notably, the increased expression of Pdr5 might contribute both to Myr efflux and to regulation of lipid flippases to control the lipid composition of membranes (Khakhina et al., 2015). Taken together, these findings indicate that SL depletion triggers significant remodeling of the PM proteome through both transcriptional and post-transcriptional regulation.

### Myr decreases the bulk inflow of the PM

**Fig. 3.**
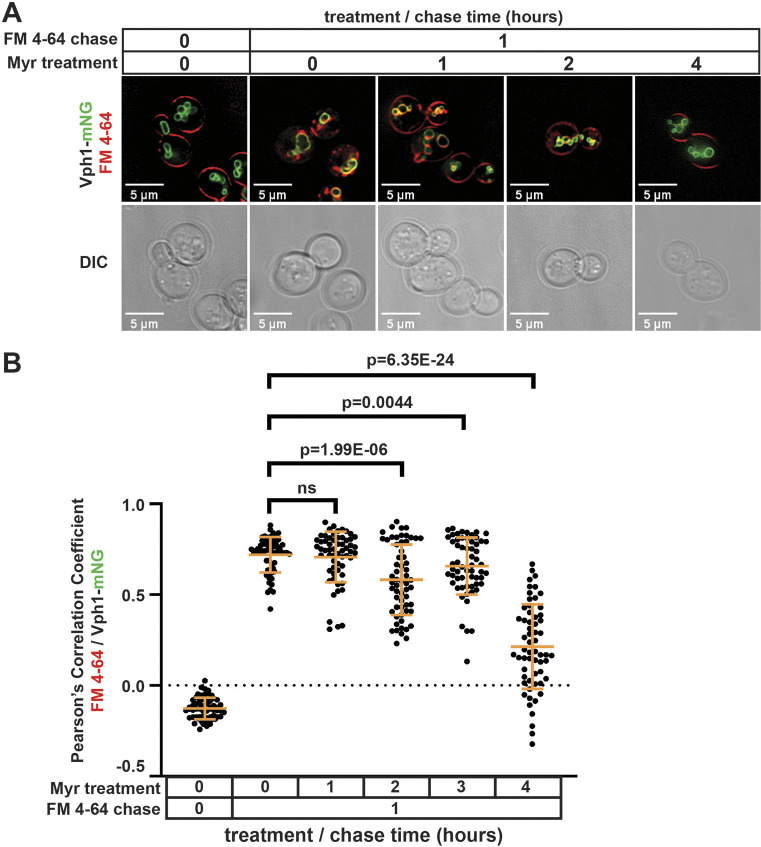
**Myr inhibits bulk endocytic trafficking of a lipophilic tracer dye.** (A) Yeast cells expressing the vacuole membrane marker Vph1–mNG (green) were pulse-labeled with the lipophilic tracer dye FM 4-64 (red) and imaged by fluorescence microscopy. Cells were treated with 400 ng ml^−1^ Myr (for 0, 1, 2 or 4 h). FM 4-64 pulse-chase times and Myr-treatment times are indicated at the top. Images are representative of duplicate experiments. (B) Colocalization of FM 4-64 and Vph1–mNG measured as Pearson's correlation coefficient values using softWoRx (v7.0.0); *n*=60 cells from duplicate experiments. Bars show the mean±s.d. ns, not significant (one-tailed paired *t*-test).

As several PM proteins accumulated at the PM following Myr treatment, we hypothesized that this might be due to a decrease in bulk endocytosis. To measure bulk endocytosis, the lipophilic tracer dye N-(3-triethylammoniumpropyl)-4-(p-diethylaminophenylhexatrienyl) pyridium dibromide (FM 4-64) was added to pulse-label the PM, followed by washing and chasing for 1 h. Bulk endocytosis results in most FM 4-64 being delivered to the limiting membrane of the vacuole (labelled by Vph1–mNG) in mock-treated cells (Fig. S4A,B). In contrast, cells treated with Myr for 4 h exhibited reduced colocalization of FM 4-64 with Vph1–mNG (Fig. 3A,B; Fig. S4C,D), indicating a significant reduction in bulk endocytosis. Notably, treatment of cells with Myr for 1 h had no effect on bulk endocytosis, whereas Myr treatment for 2 h resulted in a partial but significant defect in bulk endocytosis (Fig. 3A,B). This defect in bulk endocytosis after Myr treatment likely contributes to the surface accumulation of various PM proteins (Table 1). Given that Myr treatment impairs bulk endocytosis, it is striking that the methionine transporter Mup1 undergoes selective endocytic clearance on the same time scale (Fig. 1; Table 1). Thus, we set out to understand the mechanistic basis for selective endocytosis of Mup1 in Myr-treated cells.

### Myr-induced trafficking of Mup1 requires the Rsp5 adaptor Art2

**Fig. 4.**
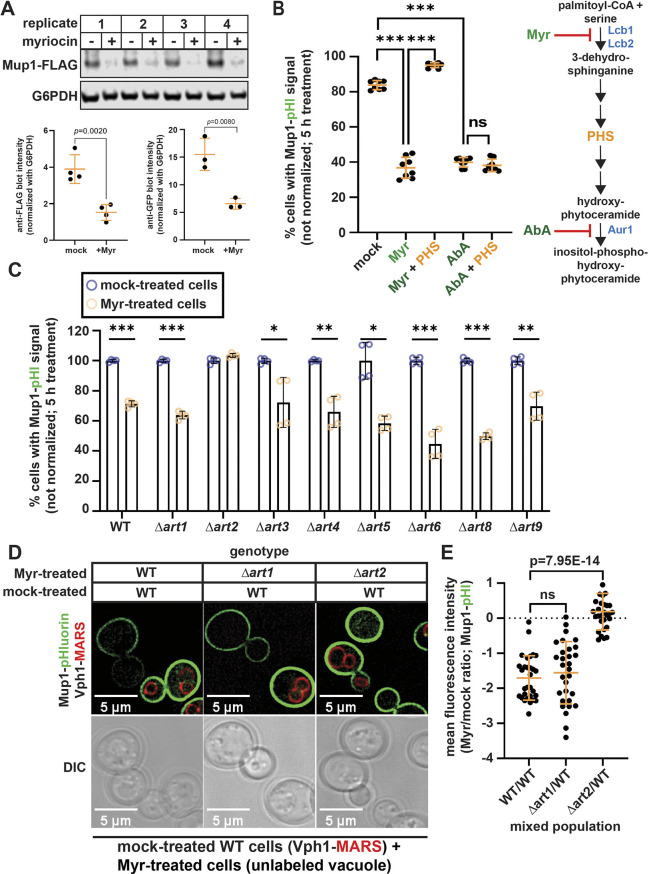
**Sphingolipid depletion induces Art2-mediated endocytic trafficking of Mup1.** (A) Myr treatment decreased the level of Mup1 (C-terminally tagged with either FLAG or GFP), as measured using western blot analysis of cell lysates. (B) Phytosphingosine (PHS) supplementation stabilized Mup1–pHluorin at the PM of Myr-treated cells but not aureobasidin A (AbA)-treated cells. Mup1–pHluorin fluorescence signal at the PM of cells was measured by a flow cytometer after 5 h of treatment (eight biological replicates; *n*=10,000 cells). The yeast sphingolipid biosynthesis pathway is shown on the right. Myr and AbA inhibit the serine palmitoyl transferase (Lcb1/Lcb2 complex) and phosphatidylinositol:ceramide phosphoinositol transferase (Aur1), respectively. (C) *ART2* is required for Myr-induced endocytic trafficking of Mup1–pHluorin. Mup1–pHluorin fluorescence signal at the PM of cells was measured by a flow cytometer after 5 h of treatment (four biological replicates; *n*=10,000 cells). (D) Mup1–pHluorin-expressing yeast cells were either mock-treated (expressing Vph1–MARS, red) or Myr-treated (unlabeled vacuoles) were mixed prior to analysis by fluorescence microscopy. Genotypes of the yeast strains used for each comparison (WT, *Δart1* and *Δart2*) are indicated on the top. Images are representative of triplicate experiments. (E) Quantification of Mup1–pHluorin fluorescence at the PM of cells shown in D. Values are ratios of mean fluorescence intensities between cell pairs in proximity to each other; *n*=30 randomly selected cell pairs from triplicate experiments. Bars show the mean±s.d. ns, not significant; *P<0.01; **P<0.001; ***P<0.0001.

To better understand how Myr triggers the selective endocytic clearance of Mup1, we first sought to validate our findings using yeast cells with endogenous Mup1 fused at its C-terminus to a 3×FLAG tag. Using quantitative immunoblotting, we found that a 5 h Myr treatment significantly decreased total levels of Mup1–FLAG (Fig. 4A). A similar result was obtained from quantitative immunoblot analysis of lysates from yeast cells expressing Mup1–GFP (Fig. 4A). Additional validation was performed using yeast cells with endogenous Mup1 fused at its C-terminus to superecliptic pHluorin, a GFP variant that does not fluoresce in the acidic environment of the vacuole (Prosser et al., 2010). In this yeast variant, steady-state levels of Mup1 at the PM can be measured by flow cytometry (Lee et al., 2019). Similar to our fluorescence microscopy results, this approach revealed that Mup1 levels at the PM began to decrease after 4 h of Myr treatment (Fig. S5A). To test whether this response was due to SL depletion, we cultured yeast cells in medium supplemented with phytosphingosine (PHS), providing an intermediate product of the SL biosynthesis pathway that effectively bypasses the Myr-imposed enzymatic block. Notably, PHS supplementation prevented the Myr-induced endocytosis of Mup1 (Fig. 4B), indicating that this response is the result of SL depletion. Similar to Myr, aureobasidin A (AbA), which inhibits the inositol phosphorylceramide synthase AUR1, also triggered the clearance of Mup1, an effect which could not be reversed by PHS supplementation (Fig. 4B). As PHS supplementation does not bypass the AbA enzymatic block, these results reveal that PM clearance of Mup1 is triggered by depletion of complex SLs that are synthesized downstream of ceramide (e.g. inositol phosphorylceramides and their mannosylated derivatives).

The endocytosis of nutrient transporters in yeast is controlled primarily by Rsp5-mediated ubiquitylation events (reviewed in Lauwers et al., 2010). Pairing of the E3 ubiquitin ligase Rsp5 with cargo adaptors called arrestin-related trafficking adaptors (ARTs) determines the specificity of substrate targeting and promotes adaptation to nutrient fluctuations and stress conditions (Kahlhofer et al., 2021; Lin et al., 2008). Analysis of the yeast transcriptional response to Myr treatment (Hepowit et al., 2023) revealed increased transcript abundance for Rsp5 and several ARTs (Fig. S5B,C), indicating that SL depletion alters gene expression in a way that could affect endocytic trafficking. To determine whether these transcript-level changes underlie alterations at the protein level, we analyzed the abundance of mNG and FLAG C-terminal fusions to various ART proteins expressed from endogenous chromosomal loci (Fig. S5D,F). Myr-induced changes in transcript and protein levels correlated in many cases [e.g. Art1 (also known as Ldb19) and Art2 (or Ecm21)] but not in every case [e.g. Art4 (or Rod1)]. Notably, both *ART1* and *ART2* were upregulated in a Myr-treatment time course (Fig. S5B,F), and both are known to regulate Mup1 endocytosis in response to excess methionine (Lin et al., 2008; MacGurn et al., 2011) and nitrogen starvation (Ivashov et al., 2020), respectively. To test whether ARTs are involved in this response, we characterized Myr-triggered Mup1–pHluorin clearance in a panel of ART deletion yeast strains and found that all ARTs tested were dispensable except for *ART2* (Fig. 4C). To validate this result, we performed fluorescence microscopy to compare the PM abundance of Mup1–pHluorin in WT, *Δart1* or *Δart2* yeast cells. Strikingly, Mup1 clearance was not detected in Myr-treated *Δart2* yeast cells, whereas loss of *ART1* did not affect this response (Fig. 4D,E). Consistent with these results, immunoblot analysis revealed that loss of Art2 stabilized Mup1–FLAG following Myr treatment (Fig. S5G,H). We next considered the possibility that SL depletion triggers recruitment of Art2 to Mup1. To test this, we used a split-Venus fluorescence complementation assay to monitor proximity between Mup1 and its adaptors. Notably, this assay revealed fluorescence complementation between Mup1 and Art1 that was independent of Myr treatment. In contrast, fluorescence complementation between Mup1 and Art2 was very low in mock conditions but significantly induced following Myr treatment (Fig. S5I,J). Taken together, these findings indicate that SL depletion triggers Art2-mediated endocytic clearance of Mup1.

Previous work demonstrated that nitrogen starvation triggered Art2-dependent endocytosis of Mup1 that required transcriptional induction of Art2 by activation of the general amino acid starvation response (Ivashov et al., 2020). This stress response requires activity of the upstream activating kinase Gcn2, which phosphorylates eIF2α to mediate the response. We hypothesized that Myr-triggered endocytosis of Mup1 might likewise occur through activation of the general amino acid starvation response and subsequent upregulation of Art2. To test this, we compared Myr-triggered Mup1 endocytosis in WT and *Δgcn2* mutant cells. Unexpectedly, we found that Gcn2 is dispensable for Myr-triggered endocytosis of Mup1 (Fig. S6A). Thus, in contrast to Mup1 endocytosis that occurs during nitrogen starvation (Ivashov et al., 2020), Myr-induced endocytosis of Mup1 occurred independently of the general amino acid starvation response. Previous work also demonstrated that Myr-mediated longevity requires amino acid-sensing pathways upstream of TORC1 (Hepowit et al., 2021), and TORC1 signaling is known to regulate endocytosis (Barthelemy et al., 2017; MacGurn et al., 2011; Merhi and André, 2012). Notably, one study reported that the Myr derivative FTY720 stimulates Gap1 endocytosis, and this was found to coincide with dysregulation of TORC1 activation (Barthelemy et al., 2017). Therefore, we decided to examine Myr-triggered Mup1 endocytosis in *Δgtr2* and *Δpib2* yeast cells, which lack upstream activators of TORC1 signaling. We found that *PIB2* and *GTR2* are both dispensable for Myr-triggered Mup1 endocytosis (Fig. S6B–E), indicating that TORC1 activation by these pathways does not promote Mup1 clearance.

### Myr-induced trafficking of Mup1 requires C-terminal K63-linked polyubiquitylation

**Fig. 5.**
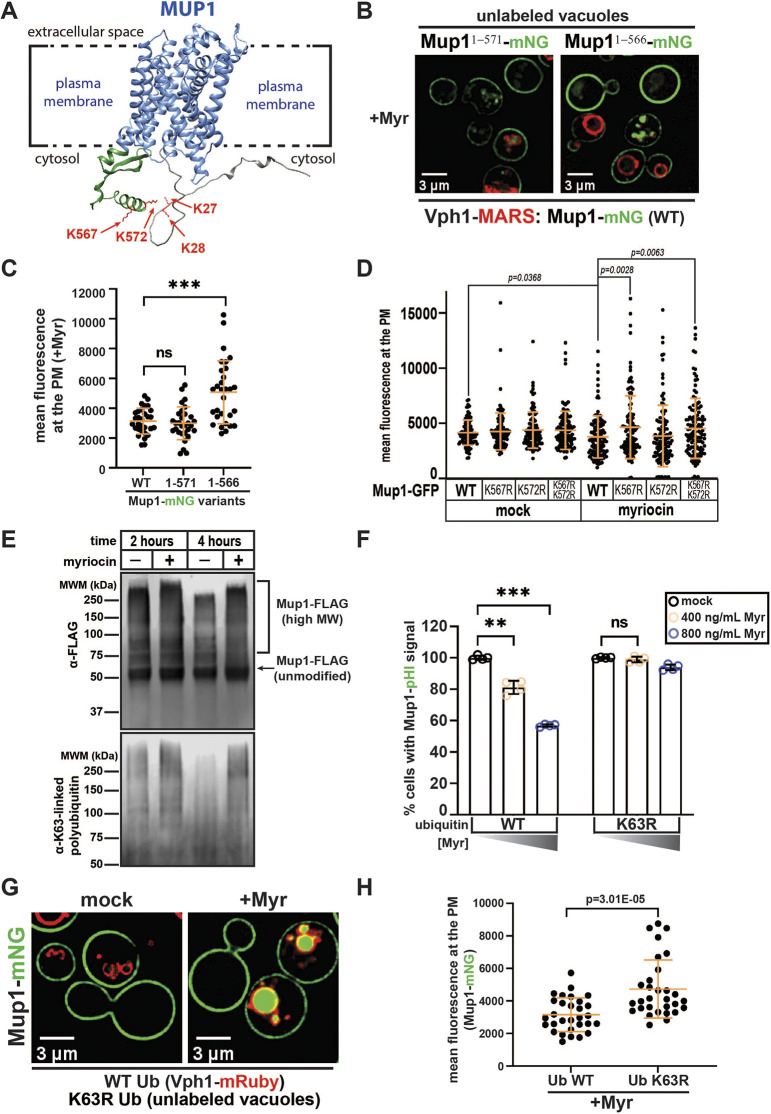
**Myr-induced trafficking of Mup1 requires K63-linked polyubiquitylation at the C-terminal lysines of Mup1.** (A) Diagram showing the predicted protein structure of Mup1 (AlphaFold). Most of the N-terminal cytosolic tail (gray) lacks predictable structure, whereas the C-terminal cytosolic tail (green) contains secondary structure elements that are predicted with higher confidence. Functional lysine residues at the N- and C-terminal cytosolic tails of Mup1 are colored in red. (B) Yeast cells expressing WT Mup1–mNG (cells with Vph1–MARS, red) or variants with C-terminal truncations (Mup1^1–571^–mNG and Mup1^1–566^–mNG) were visualized by fluorescence microscopy after treatment with Myr (400 ng ml^−1^) for 5 h. Images are representative of triplicate experiments. (C) Quantification of Mup1–mNG expressed as mean fluorescence intensity at the PM of cells visualized in B (*n*=30 cells from triplicate experiments). (D) Mup1–GFP variants (Mup1^WT^, Mup1^K567R^, Mup1^K572R^ and Mup1^K567R,K572R^) were expressed exogenously from a plasmid. Mean fluorescence intensity levels of Mup1–GFP at the PM (*n*=94–121 cells from duplicate experiments) were quantified following mock treatment or treatment with Myr (400 ng ml^−1^) for 5 h. (E) Anti-FLAG and anti-K63-linked polyubiquitin immunoblots of Mup1–FLAG enriched by anti-FLAG immunoprecipitation after 2 or 4 h of Myr treatment. Images are representative of duplicate experiments. MW, molecular weight; MWM, molecular weight marker. (F) Mup1–pHluorin trafficking was analyzed by flow cytometry of yeast cells expressing either WT or K63R ubiquitin from a single source. Mup1–pHluorin levels were measured after 5 h treatment with or without Myr (four biological replicates; *n*=10,000 cells). (G) Yeast cells expressing Mup1–mNG (green) and either WT ubiquitin (labeled with Vph1–mRuby in red) or K63R ubiquitin (unlabeled vacuoles) were mixed and imaged by fluorescence microscopy following mock or Myr treatment. Images are representative of triplicate experiments. (H) Quantification of Mup1–mNG mean fluorescence intensity at the PM of cells shown in G. *n*=30 cells. Bars show the mean±s.d. ns, not significant; **P<0.001; ***P<0.0001 (one-tailed paired *t*-test).

Ubiquitylation at N-terminal lysines (K27 and K28) is required for Mup1 endocytosis in response to excess methionine (Busto et al., 2018; Ghaddar et al., 2014; Guiney et al., 2016), whereas ubiquitylation at C-terminal lysines (K567 and K572) is reported to mediate endocytic clearance in response to nitrogen starvation (Ivashov et al., 2020). Structure predictions from the AlphaFold protein structure database (Jumper et al., 2021) indicate that the N-terminal ubiquitylation sites (K27 and K28) exist in a largely unstructured region, whereas the C-terminal ubiquitylation sites (K567 and K572) occur in an α-helical region (Fig. 5A). As Art1-mediated ubiquitylation of Mup1 occurs at N-terminal lysines (K27 and K28) and Art2 was previously reported to bind to the C-terminus of Mup1 (Ivashov et al., 2020) we predicted that ubiquitylation of C-terminal lysine residues might be required for Myr-induced endocytosis of Mup1. To test this prediction, we characterized Myr-induced trafficking of Mup1–mNG in strains with short C-terminal truncations lacking one or both C-terminal lysine residues (Mup1^1–571^ and Mup1^1–566^, respectively). Whereas Mup1^1–571^–mNG exhibited Myr-induced endocytic clearance, Mup1^1–566^–mNG was unresponsive to Myr treatment (Fig. 5B,C). To further test this prediction, we exogenously expressed Mup1–GFP variants (Mup1^WT^, Mup1^K567R^, Mup1^K572R^, and Mup1^K567R,K572R^) in yeast cells and analyzed PM abundance in response to Myr treatment. We observed a Myr-triggered decrease in PM abundance of Mup1^WT^ and Mup1^K572R^, whereas Mup1^K567R^ and Mup1^K567R,K572R^ were not responsive to Myr treatment (Fig. 5D). These results suggest that Myr-triggered Mup1 endocytosis requires ubiquitylation at K567.

To measure the ubiquitylation of Mup1, we affinity-purified Mup1–FLAG from yeast lysates and analyzed it by SDS-PAGE and quantitative immunoblotting. This analysis revealed a significant increase in K63-linked ubiquitin (Ub) polymers associated with Mup1 in response to Myr treatment (Fig. 5E). This increase in K63-linked ubiquitin polymers associated with Mup1 was found to be Art1 independent but Art2 dependent (Fig. S6F). Previous work demonstrated that conjugation to monoubiquitin is sufficient for Mup1 endocytosis in response to excess methionine (Stringer and Piper, 2011). In contrast, we found that yeast cells expressing Ub^K63R^ as the sole source of ubiquitin were deficient for Myr-triggered endocytosis of Mup1 (Fig. 5F–H; Fig. S6G), indicating that K63-linked ubiquitin polymers are required for this response. Taken together, our results reveal that Myr-induced endocytosis of Mup1 is mediated by ubiquitin but proceeds by a mechanism that is distinct from methionine-induced endocytosis.

### Ede1 and Ent1 function redundantly in Myr-mediated Mup1 endocytic clearance

**Fig. 6.**
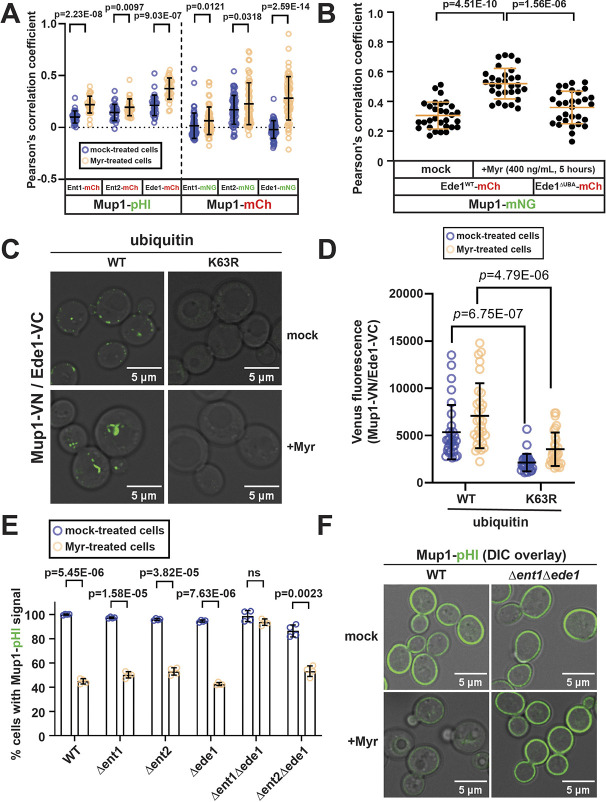
**Myr-induced trafficking of Mup1 requires the endocytic adaptor proteins Ent1 and Ede1.** (A) Yeast cells expressing the indicated fluorescent protein fusions were analyzed by fluorescence microscopy following 5 h of mock treatment or Myr treatment. Colocalization was measured using Pearson's correlation coefficient (*n*=30–60 cells from triplicate experiments). (B) Colocalization between Ede1–mCherry and Mup1–mNG was analyzed by measuring Pearson's correlation coefficients (*n*=30 cells from triplicate experiments) for the indicated conditions. (C) A split-Venus fluorescence complementation assay with the N-terminal fragment of Venus fused to Mup1 (Mup1–VN) and the C-terminal fragment of Venus fused to Ede1 (Ede1–VC) was used to analyze proximity between Mup1 and Ede1. Cells expressing either WT or K63R ubiquitin were analyzed by fluorescence microscopy after 5 h of mock treatment or Myr treatment, as indicated. Images are representative of triplicate experiments. (D) Total Venus fluorescence (Mup1–VN and Ede1–VC) of cells analyzed from the experiment shown in C (*n*=30 cells from triplicate experiments). (E) Myr-triggered Mup1–pHluorin trafficking was analyzed by flow cytometry in yeast cells with the indicated genotypes. Mup1–pHluorin fluorescence was measured in cells after 5 h with or without Myr treatment (four biological replicates; *n*=10,000 cells). (F) Fluorescence microscopy of WT and *Δent1Δede1* yeast cells expressing Mup1–pHluorin (green) after 5 h with or without Myr treatment. Images are representative of triplicate experiments. Bars show the mean±s.d. ns, not significant (one-tailed paired *t*-test).

Multiple ubiquitin-binding proteins in yeast, including the epsins Ent1 and Ent2 and the epsin-like protein Ede1, function as adaptors that capture ubiquitylated cargo during endocytic vesicle formation. Among these, Ede1 was previously found to be crucial in mediating Mup1 trafficking in response to excess methionine (Wrasman et al., 2018). To examine the role of endocytic adaptors in the cellular response to Myr, we analyzed the abundance of endocytic adaptors fused to mNG at the C-terminus (expressed from endogenous chromosomal loci) using total fluorescence measurements. This analysis revealed significant increases in protein levels of Ent1 and Ede1 by 3 h of Myr treatment, and a slight increase in the levels of Ent2 by 5 h of Myr treatment (Fig. S7A). Notably, the subcellular localization of Ent1–mNG, Ent2–mNG and Ede1–mNG appeared to be unaffected by Myr treatment (Fig. S7B). We next measured colocalization between endocytic adaptors and Mup1 in response to Myr treatment. This analysis revealed that Myr treatment induced colocalization of Mup1 with Ent1 and Ede1, and to a lesser extent with Ent2 (Fig. 6A). Importantly, we did not observe significant changes for Ede1 colocalization with either Can1 (an arginine transporter) or Pil1 (an eisosome component) during the same time course (Fig. S7C). To determine whether the increased association between Mup1 and Ede1 was due to ubiquitin binding, we analyzed colocalization between Mup1–mNG and a variant of Ede1 lacking its C-terminal UBA domain, which is known to preferentially interact with K63-linked polymers (Sims et al., 2009). Notably, the Ede1^Δuba^ variant did not exhibit increased association with Mup1 in response to Myr treatment (Fig. 6B). To determine whether this association requires K63-linked ubiquitin polymers, we used a split-Venus fluorescence complementation assay to monitor proximity between Mup1 and Ede1. Fluorescence complementation between Mup1 and Ede1 was observed in mock-treated cells and significantly increased following Myr treatment (Fig. 6C,D). In both conditions, fluorescence complementation was significantly decreased in yeast cells expressing Ub^K63R^ as the sole source of ubiquitin (Fig. 6C,D). These results suggest that SL depletion promotes association between Mup1 and endocytic adaptors, likely mediated through the formation of K63-linked ubiquitin polymers.

We next sought to determine whether any of these endocytic adaptors are required for Myr-triggered endocytosis of Mup1. We found that Ent1, Ent2 and Ede1 were all individually dispensable for Myr-induced Mup1 trafficking (Fig. 6E), despite the fact that loss of Ede1 was sufficient to prevent methionine-triggered Mup1 endocytosis (Fig. S7D) (Wrasman et al., 2018). Analysis of double mutants revealed that Myr-triggered Mup1 endocytosis occurred normally in *Δent2Δede1* cells but was blocked in *Δent1Δede1* cells (Fig. 6E,F; Fig. S7E). Notably, *Δent1Δent2* double-mutant yeast cells are inviable (Wrasman et al., 2018) and thus could not be tested in our analysis. These data reveal that Ent1 and Ede1 are redundant with respect to Myr-triggered Mup1 endocytic clearance.

## DISCUSSION

It is well established that genetic and pharmacological interventions that perturb SL biosynthesis promote longevity in a variety of model organisms (Huang et al., 2014), although how SL homeostasis and life span are coupled is not fully understood. Previously, we have reported that SL reduction in yeast extends chronological life span (Huang et al., 2012) and that this is associated with a state of amino acid restriction, which is accomplished at least in part by decreasing the uptake of extracellular amino acids (Hepowit et al., 2021). Here, we report the unexpected result that, for almost all nutrient transporters and PM-associated proteins examined, Myr treatment either had no effect or increased abundance at the PM (Table 1). The only exception was Mup1, which undergoes endocytic clearance following 4 h of Myr treatment (Fig. 3; Fig. S4). Thus, the observed decrease in abundance of most amino acids observed upon Myr treatment is not likely owing to broad endocytic clearance of AATs, and in fact occurs despite the increased PM abundance of Bap3 and Tat2. Similar results were observed for hexose transporters, some of which accumulated at the PM despite decreased glucose uptake during a Myr-treatment time course. One possible explanation for this apparent disparity is that SL depletion might lower transport activity without inducing endocytosis. In some cases, the accumulation of specific PM proteins following Myr treatment might be due to inhibition of bulk endocytosis, which was observed using FM 4-64-trafficking assays (Fig. 3; Fig. S4). Alternatively, it is also possible that cells respond to decreased amino acid and glucose availability by upregulating the biosynthesis and secretion of specific nutrient transporters. Our analysis suggests that the activities of many nutrient transporters are coupled to SL abundance at the PM, and future studies will be needed to address the mechanism of this coordination.

**Fig. 7.**
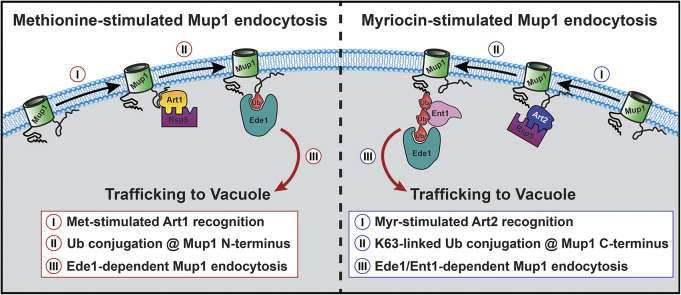
**Comparison of Mup1 endocytic trafficking as stimulated by excess methionine (left) or sphingolipid depletion (right).** Methionine stimulates Art1-dependent ubiquitin modification of the N-terminus of Mup1, which then undergoes endocytosis mediated by the endocytic adaptor Ede1. In contrast, myriocin stimulates Art2-dependent ubiquitin modification of the C-terminus of Mup1, endocytosis of which requires K63-linked ubiquitin polymers and the endocytic adaptors Ent1 and Ede1.

The methionine transporter Mup1 was unique amongst PM proteins in being selectively targeted for endocytic clearance following SL depletion. More specifically, our finding that both Myr and AbA triggered Mup1 endocytosis, but addition of PHS only suppressed the effect of Myr and not the effect of AbA, indicates that depletion of inositol phosphorylceramides (and/or downstream products) triggers Mup1 endocytosis. The mechanism of methionine-induced endocytosis of Mup1 is well characterized (Guiney et al., 2016; Lin et al., 2008; Stringer and Piper, 2011; Wrasman et al., 2018): (1) it involves ubiquitylation of N-terminal lysine residues by the Art1–Rsp5 E3 ubiquitin ligase complex, (2) it occurs independently of ubiquitin polymer formation, and (3) it requires the endocytic adaptor Ede1. In contrast, we found that Myr-induced endocytosis of Mup1 (1) is mediated by the Art2–Rsp5 E3 ubiquitin ligase complex, (2) requires C-terminal lysine residues, (3) requires the formation of K63-linked ubiquitin polymers, and (4) requires either Ede1 or Ent1 as an endocytic adaptor. Furthermore, Myr treatment induces colocalization of Mup1 with Ent1 and Ede1, and the Mup1-Ede1 colocalization requires its C-terminal UBA domain, which is known to interact with ubiquitin. Thus, Myr-triggered Mup1 endocytosis is mechanistically distinct from methionine-mediated Mup1 endocytosis (Fig. 7).

Importantly, Mup1 endocytosis has also been reported to occur during cellular adaptation to other stresses and environmental changes. Endocytosis of many PM proteins, including Mup1, occurs in response to depletion of nicotinic acid by a mechanism that relies on tetraspan Cos proteins but is distinct from known ART–Rsp5 complexes (MacDonald et al., 2015). Another recent study reported that nitrogen starvation triggers endocytosis of multiple AATs, including Mup1, Can1, Lyp1 and Tat2, as well as glucose transporters, such as Hxt1, Hxt2 and Hxt3 (Ivashov et al., 2020). The nitrogen starvation-induced endocytosis of Mup1 and several other AATs (Can1, Lyp1 and Tat2) was Art2 dependent and required Gcn2-dependent induction of Art2 expression (Ivashov et al., 2020). Although both nitrogen starvation and SL depletion induce Art2-dependent endocytosis of Mup1, there are two notable mechanistic distinctions. First, although nitrogen starvation induces Art2-mediated endocytosis of multiple nutrient transporters, the endocytosis induced by SL depletion is very selective for Mup1. Indeed, some nutrient transporters like Tat2, Hxt1 and Hxt2 were internalized during nitrogen starvation but accumulated at the PM in response to SL depletion. Second, although Gcn2 is required for Mup1 endocytosis during nitrogen starvation (Ivashov et al., 2020), it is dispensable for Myr-triggered Mup1 endocytosis (Fig. S6A). These distinctions indicate that Art2 is broadly activated in a Gcn2-dependent manner in response to nitrogen starvation, but that its Gcn2-independent activation during SL depletion is restricted to Mup1. Taken together, these results reveal distinct PM remodeling processes that occur during cellular adaptation to nitrogen starvation or to SL depletion.

It remains unclear why the methionine transporter Mup1 is selectively targeted for endocytic clearance following SL depletion. One possibility is that Mup1 transmembrane domains interact with SLs to promote its stability or organization. Indeed, there is a precedent for SL interactions with transmembrane domains (Contreras et al., 2012) and such interactions could regulate the function and stability of Mup1. There is also a precedent for SL-dependent organization of PM proteins: Slm1 was found to associate with eisosome domains at the PM in a manner that is dependent on SLs (Berchtold et al., 2012). Based on these observations, we hypothesize that interactions between Mup1 and SLs help stabilize Mup1 and promote its transport function, and loss of these interactions is a destabilizing event sufficient to trigger recognition and clearance.

The results of this study shed light on a cellular response for specific removal of methionine transporters from the cell surface. The cellular response to Myr treatment is associated with enhanced life span, and recent reports have linked this longevity effect to decreased methionine and Mup1 clearance (Hepowit et al., 2023, 2021). Indeed, stabilizing Mup1 at the PM prevented Myr-mediated longevity in yeast (Hepowit et al., 2023), underscoring the importance of Mup1 endocytosis for aging. This is consistent with a recent study that reported that decreased intracellular methionine concentration mediates life span extension associated with caloric restriction (Zou et al., 2020). Collectively, these studies highlight the critical importance of methionine metabolism as a determinant of aging, and they suggest commonalities between life span extension associated with caloric restriction and SL depletion. Ultimately, improved understanding of cellular adaptation to SL depletion, particularly with respect to PM transport functions that regulate intracellular nutrient concentrations, will reveal how compounds like Myr promote health and longevity.

## MATERIALS AND METHODS

### Strains, media and growth conditions

*Saccharomyces cerevisiae* strains (Table S1) expressing endogenous reporter proteins fused with fluorescent proteins were generated by homologous recombination or mating. Cells in synthetic complete dextrose (SCD) medium were grown at 26°C with agitation (220 rpm) to mid-log phase (OD_600_=0.3–0.6) and treated with 400 ng ml^−1^ Myr (Cayman Chemical Company), AbA (TaKaRa), phytosphingosine (Tokyo Chemical Industry) or mock solution (95% ethanol) as needed. Cells expressing Hip1–mNG were cultured in low-histidine SCD (2 µg ml^−1^ histidine), whereas strains expressing Hxt6–mNG and Hxt7–mNG were grown in low-glucose SCD (0.2% glucose).

### Fluorescence microscopy

Yeast cells endogenously expressing fluorescent fusion proteins (mNG, MARS or mCherry) were grown to mid-log phase in the indicated SCD broth, treated with Myr for 5 h, concentrated by centrifugation (3500 ***g*** for 10 s) and visualized using a DeltaVision Elite Imaging system [Olympus IX-71 inverted microscope; Olympus 100× oil objective (1.4 NA); DV Elite sCMOS camera, GE Healthcare]. For bulk endocytosis experiments using FM 4-64 (Invitrogen), cells were prepared as previously described (Tumolo et al., 2020). Fluorescence colocalization of superimposed red and green channels was measured using Pearson's correlation coefficients in an unbiased, automated thresholding as analyzed by the softWoRx software (v7.0.0; GE Healthcare). Images obtained from the red (Alexa Fluor 594; 475 nm excitation, 523 nm emission) and green (FITC; 575 nm excitation, 632 nm emission) filter channels were deconvolved using softWoRx, and the mean fluorescence intensity units at the PM were measured using Fiji (Schindelin et al., 2012). For proximity imaging, we used a split-Venus system by fusing Venus fragments (VN and VC) to proteins of interest (endogenous expression of Mup1–VN with Art1–VC, Art2–VC or Ede1–VC). Proximity was indicated by complementation of Venus fluorescence as detected by fluorescence microscopy.

### Flow cytometry

The endocytic trafficking of Mup1–pHluorin was analyzed using a flow cytometer as previously described (Hepowit et al., 2021; Lee et al., 2019) with minor modifications. Briefly, cells endogenously expressing Mup1–pHluorin were grown to mid-log phase, treated with Myr or AbA, or in combination with 2 μM phytosphingosine (PHS) (Tokyo Chemical Industry) for 5 h, and analyzed using the BD Accuri C6 Plus Flow Cytometer. The flow was set in fast fluidics and the relative intensity of Mup1–pHluorin in 10,000 cells was measured in the FITC channel using 90% histogram gating of the mock-treated cells for signal normalization.

### Western blotting

Endogenously expressed Mup1–FLAG was isolated by anti-FLAG immunoprecipitation as previously described (Hepowit et al., 2020), dissolved in urea sample buffer containing 10% β-mercaptoethanol, and resolved in 12% Bis-Tris PAGE gels by electrophoresis. Proteins were transferred onto PVDF membranes (0.45 µm, GE Healthcare Amersham) by electrophoretic transblotting, blocked with 3% bovine serum albumin, and incubated with the following primary antibodies: mouse anti-FLAG (1:1000; Sigma-Aldrich; clone M2; F3165; RRID AB_262044), mouse anti-ubiquitin (1:10,000; LifeSensors; clone VU-1; VU101; RRID AB_2716558), rabbit anti-K63-linked ubiquitin (1:1000; EMD Millipore; clone apu3; 05-1308; RRID AB_1587580) and rabbit anti-G6PDH (1:10,000; Sigma-Aldrich; A9521; RRID AB_258454). The secondary antibodies used were IRDye 680RD-goat anti-mouse (LI-COR; 926–68070; RRID AB_10956588) and IRDye 800CW-goat anti-rabbit (LI-COR; 926–32211; RRID AB_621843) IgGs. Fluorescence of blots was visualized using the Odyssey CLx Imaging System (LI-COR) and quantified using Image Studio Lite (LI-COR).

### Radiolabeled glucose uptake assay

Cells were grown in triplicate for 24 h at 30°C in SDC medium (prepared as per Fabrizio and Longo, 2007) with shaking. To initiate an uptake assay, 4.8 OD_600_ units of cells were added to a 125 ml flask containing 25 ml of SDC medium with and without 575 ng/ml myriocin (final concentration of ethanol in all flasks was 0.3%) and incubated in a 30°C water bath shaker. Uptake assays were performed at the time points indicated in Fig. 2A. Three OD_600_ units of cells were filtered on a nylon filter (VWR 28159-744, HAWP025000, 25 mm diameter) and washed twice with 5 ml of 0.1 M KPO_4_ (pH 6.5, warmed to 30°C). The filter was placed on the inside of a sterile 50 ml conical centrifuge tube containing 0.995 ml of uptake mix (100 mM phosphate, pH 6.5, 100 mM glucose plus 5 μl of ^3^H-glucose {D-[6-^3^H(N)], specific activity 60 Ci/mM, 5 mCi/5 ml, American Radiolabeled Chemicals, St. Louis, MO, USA}). Tubes were vortexed for 15 s to start isotope uptake and placed in a 30°C water bath with intermittent mixing. After 4 min, 0.75 ml of sample was transferred to a glucose-saturated filter and washed twice with 5 ml of ice-cold Nanopure water. Filters were transferred to 4 ml of Ultima Gold LSC Cocktail (Millipore Sigma) and counted in a TRI-CARB 4810TR Liquid Scintillation Counter (PerkinElmer). Radioisotope background counts on filters was determined by filtering 0.75 ml of uptake assay mix and washing twice. Specific activities were determined by using a standard curve of mg protein/OD_600_ units versus time of cell growth (0–6 h), where protein concentration was determined by a Bio-Rad Bradford assay.

### RNA-sequencing data analysis

All RNA-sequencing data presented in this paper was mined from previously published data (Hepowit et al., 2023). For this, the authors isolated total RNA from the lysate of five OD_600_ units of cells, sampled every hour of Myr treatment (up to 6 h), using the RNAeasy Mini Kit (QIAGEN). RNA samples were frozen in dry-ice ethanol bath and stored at −80°C until use. RNA sequencing was performed at the J. Carver Biotechnology Center at the University of Illinois and data were deposited in the Gene Expression Omnibus (GSE199904).

## Supplementary Material

10.1242/joces.260675_sup1 Supplementary information
