# Supplementary material for: Art2 mediates selective endocytosis of methionine transporters during adaptation to sphingolipid depletion
Source: J Cell Sci. 2023 Jul 25;136(14):jcs260675. doi: 10.1242/jcs.260675 (PMC10399987; doi:10.1242/jcs.260675)
Supplement: Supplementary information [file joces-136-260675-s1.pdf]

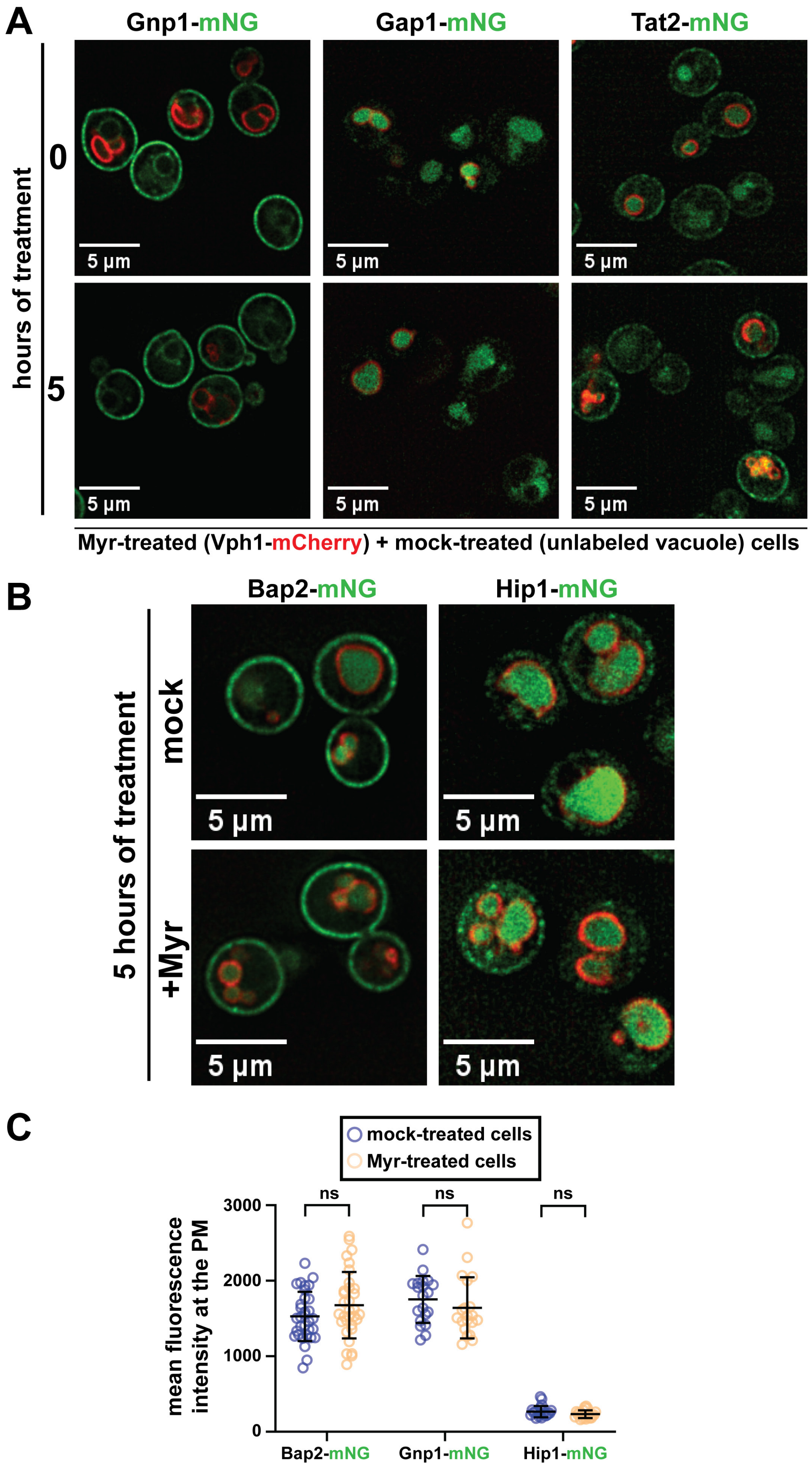

Journal of Cell Science • Supplementary information

Fig. S1. Microscopy of cells expressing endogenous mNG-tagged amino acid transporters treated with or without Myr.

(A) Yeast cells expressing the indicated amino acid transporters (AATs) with a C-terminal mNG tag (green) were visualized under a fluorescence microscope after treatment with Myr (400 ng·mL^{-1}) or mock solution (95% ethanol). Myr-treated cells expressed mCherry-tagged Vph1 (a vacuolar marker, red) and mock-treated cells have unlabeled vacuoles.

(B) Yeast cells expressing the indicated AAT C-terminal mNG fusion after 5 hours of mock treatment or Myr treatment. Vph1-mCherry (red) is used as a vacuolar marker.

(C) Quantification of the mean fluorescence intensity of the indicated AAT-mNG at the PM, measured using Fiji (n = 20 cells; ±SD (error bars)).

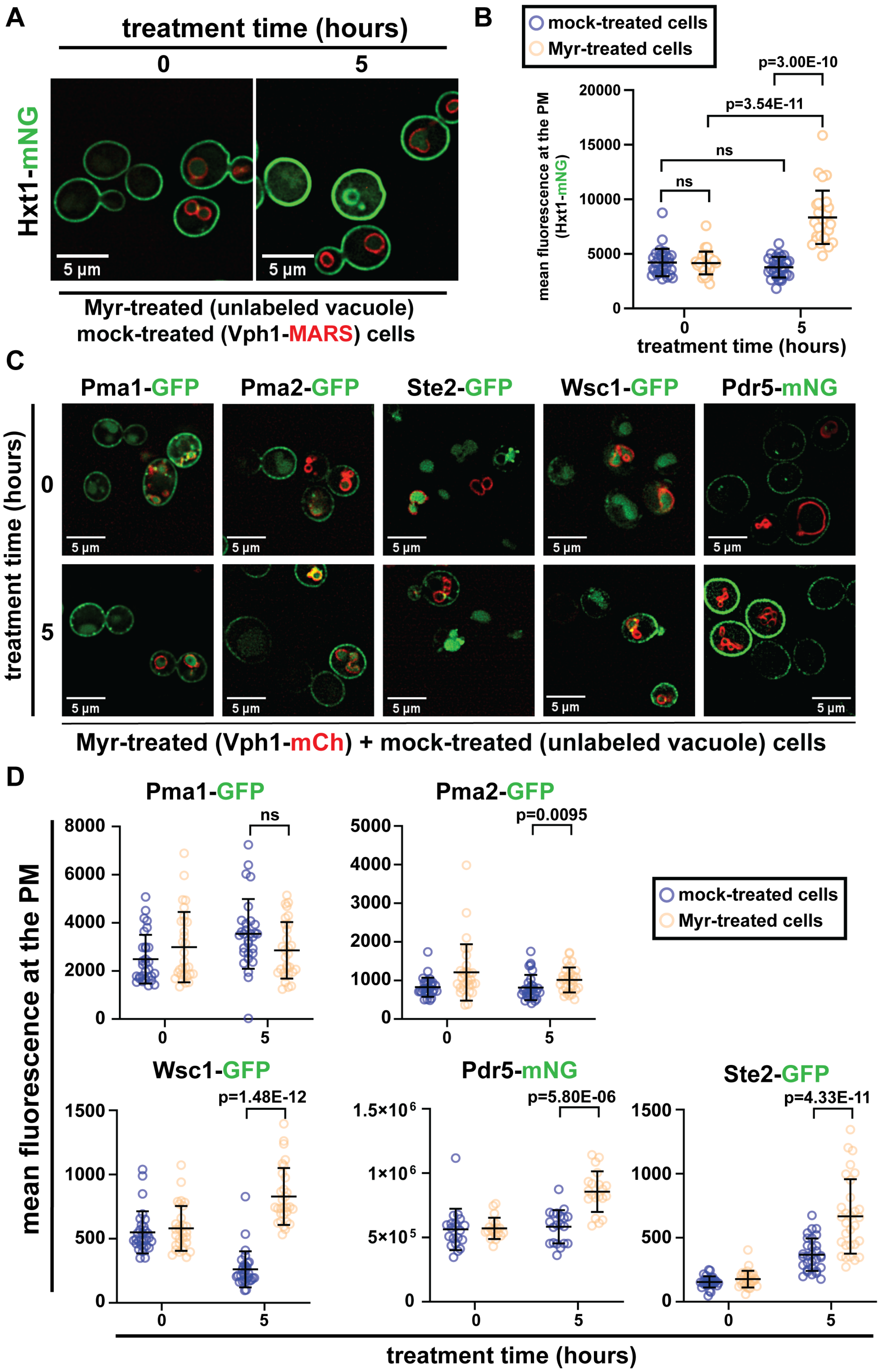

Journal of Cell Science • Supplementary information

Fig. S2. Myr stabilizes a subset of integral proteins at the PM.

(A) Yeast cells expressing endogenous Hxt1-mNG treated with or without Myr in a mixed population microscopy assay. Mock-treated cells express Vph1-MARS as a red marker of vacuolar membranes, while Myr-treated cells have unlabeled vacuoles.

(B) Total fluorescence intensity of Hxt1-mNG at the PM of cells shown in FIG S2A (n = 20 cells; ±SD (error bars)).

(C) Yeast cells expressing the indicated PM proteins with a C-terminal GFP or mNG tag (green) were visualized under a fluorescence microscope after treatment with Myr (400 $ng \cdot mL^{-1}$) or mock solution (95% ethanol). Myr-treated cells expressed mCherry-tagged Vph1 (a vacuolar marker, red) and mock-treated cells have unlabeled vacuoles.

(D) Mean fluorescence intensity of mNG or GFP signal at the PM of cells shown in FIG 2C (n = 20-30 cells; ±SD (error bars)).

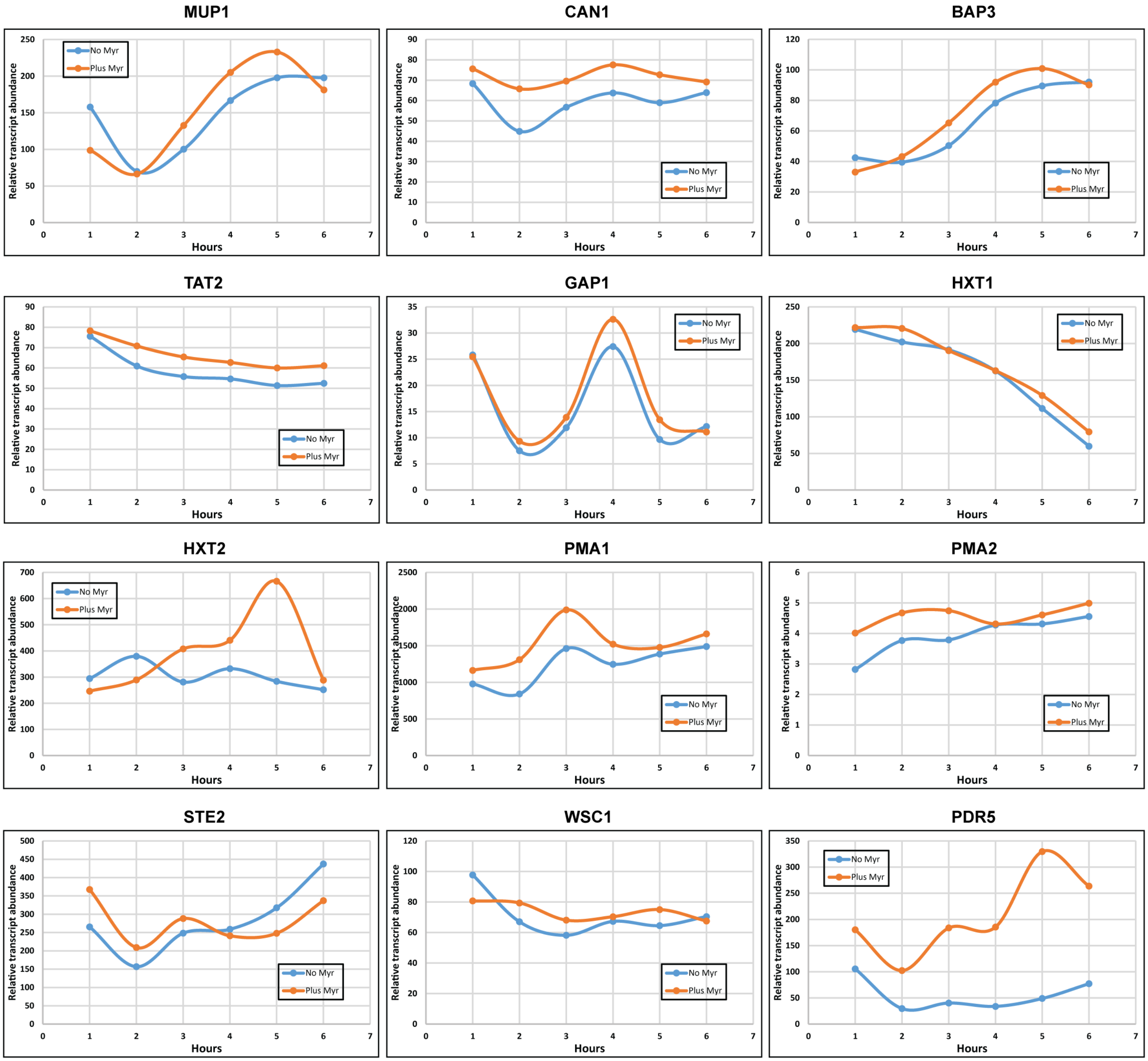

Fig. S3. Analysis of transcripts encoding PM proteins over a mock treatment (blue) or Myr treatment (orange) time course. The data presented was mined from a recent study reporting on the yeast transcriptional response to Myr treatment [23].

Journal of Cell Science • Supplementary information

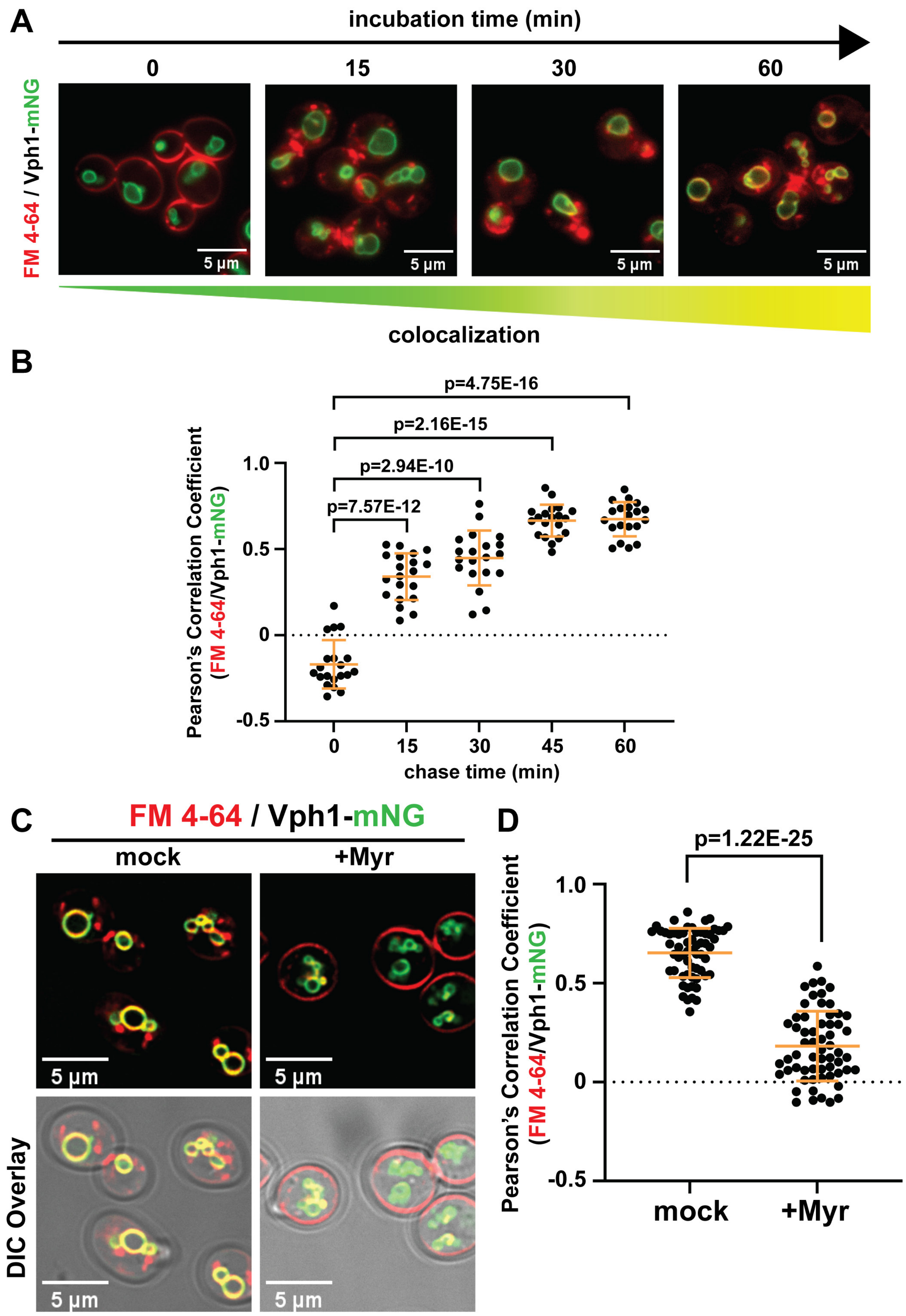

Journal of Cell Science • Supplementary information

Fig. S4. Myr inhibits bulk endocytic trafficking of a lipophilic tracer dye.

(A) Pulse-chase analysis of the lipophilic tracer dye FM 4-64 (fluorescent red) was performed in yeast expressing the vacuolar membrane marker Vph1-mNG (fluorescent green). Yellow coloration indicates the colocalization of FM 4-64 and Vph1-mNG at the vacuole.

(B) Quantification of colocalization between FM 4-64 and Vph1-mNG at different chase times (0, 15, 30, 45 or 60 min). Colocalization is expressed as Pearson's Correlation Coefficient values (n = 20 cells; ±SD (error bars)) measured using softWorx (ver. 7.0.0).

(C) Fluorescence microscopy of Myr-treated *S. cerevisiae* BY4741 cells (4 hours) after one hour incubation with FM 4-64.

(D) Quantification of Fm4-64/Vph1-mNG colocalization on Myr-treated cells (Suppl. Fig. 3C) after incubation with FM 4-64 for one hour. Colocalization expressed as Pearson's Correlation Coefficient values (n = 60 cells; ±SD (error bars)) measured using softWorx (ver. 7.0.0).

Journal of Cell Science • Supplementary information

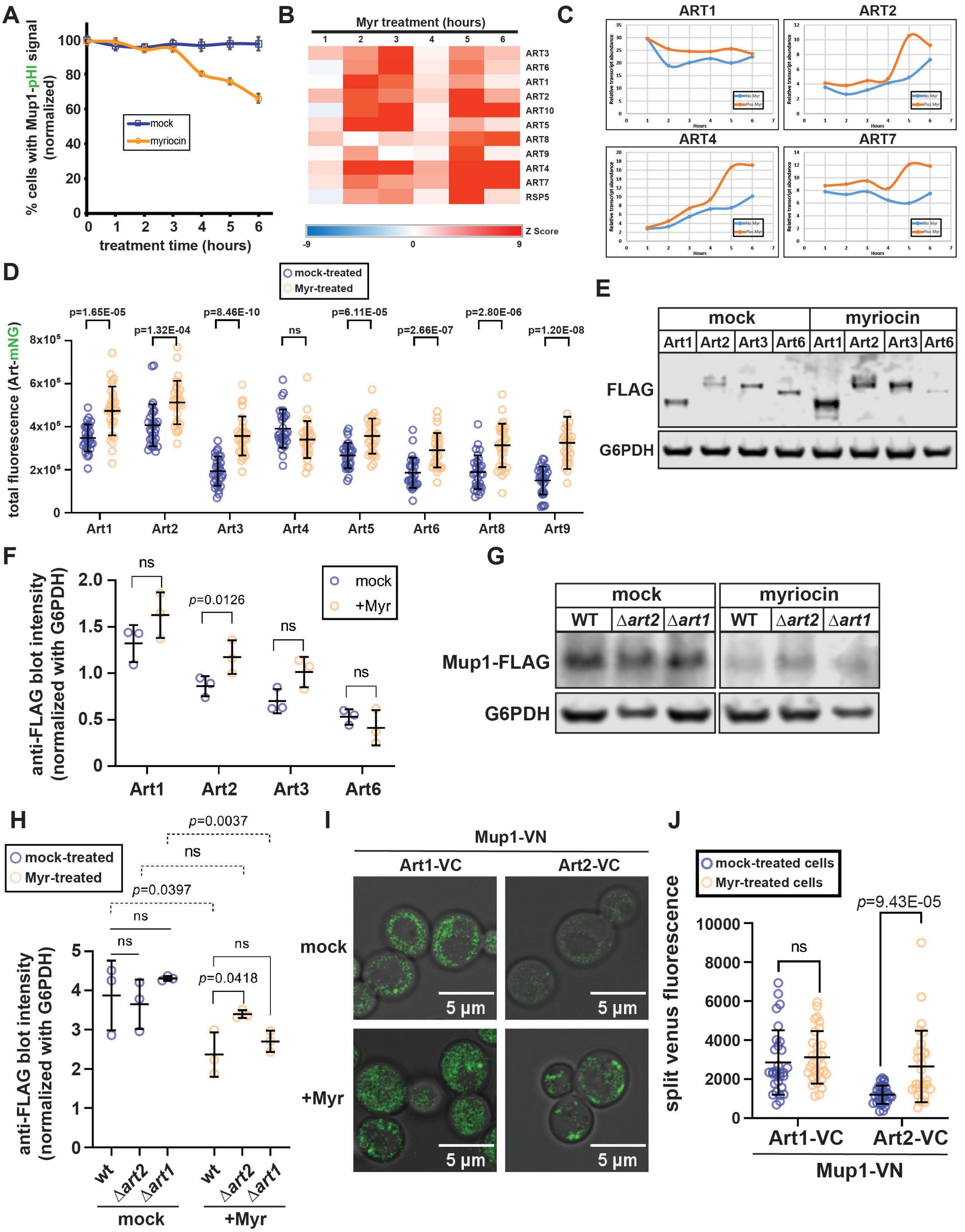

Journal of Cell Science • Supplementary information

Fig. S5. Myr treatment triggers Art2-dependent endocytic clearance of Mup1.

(A) Mup1-pHluorin fluorescence was monitored by flow cytometry in a mock-treatment or Myr-treatment time course (four biological replicates; n = 10,000 cells for each biological replicate; ±SD (error bars)).
(B) Analysis of transcript levels of yeast arrestin-like trafficking adaptors (ARTs) and the E3 ubiquitin ligase Rsp5 in a Myr treatment time course. This analysis was performed using previously published RNAseq data [23].

(C) Transcript levels of ART1, ART2, ART4, and ART7 in cells treated with or without Myr as determined by RNAseq. Data was mined from published RNAseq data [23].
(D) Analysis of expression levels of different ART proteins in mock-treated and Myr-treated cells. Yeast strains harboring endogenous C-terminal mNG tags for the indicated ART proteins were measured for total mNG fluorescence.
(E) Analysis of expression levels of different ART proteins after 5 hours of either mock-treatment or Myr-treatment. Yeast strains harboring endogenous C-terminal 3xFLAG tags for the indicated ART proteins were analyzed by immunoblot. G6PDH was used as a loading control.
(F) Quantification of results shown in FIG S5E. Anti-FLAG immunoblot intensity was quantified and normalized to G6PDH (from three biological replicates).
(G) Mup1-FLAG levels were analyzed by immunoblot in mock-treated and Myr-treated yeast cells of the indicated genotypes. G6PDH was used as a loading control.
(H) Quantification of results shown in FIG S5G. Anti-FLAG immunoblot intensity was quantified and normalized to G6PDH (from three biological replicates).
(I) Split venus fluorescence complementation analysis in cells expressing Mup1-VN and Art1-VC or Art2-VC. Cells treated for 5 hours with mock-treatment or Myr-treatment were analyzed by fluorescence microscopy.
(J) Quantification of the results shown in FIG S5I. Total venus fluorescence was measured for 30 randomly selected cells after 5 hours of either mock-treatment or Myr-treatment.

Journal of Cell Science • Supplementary information

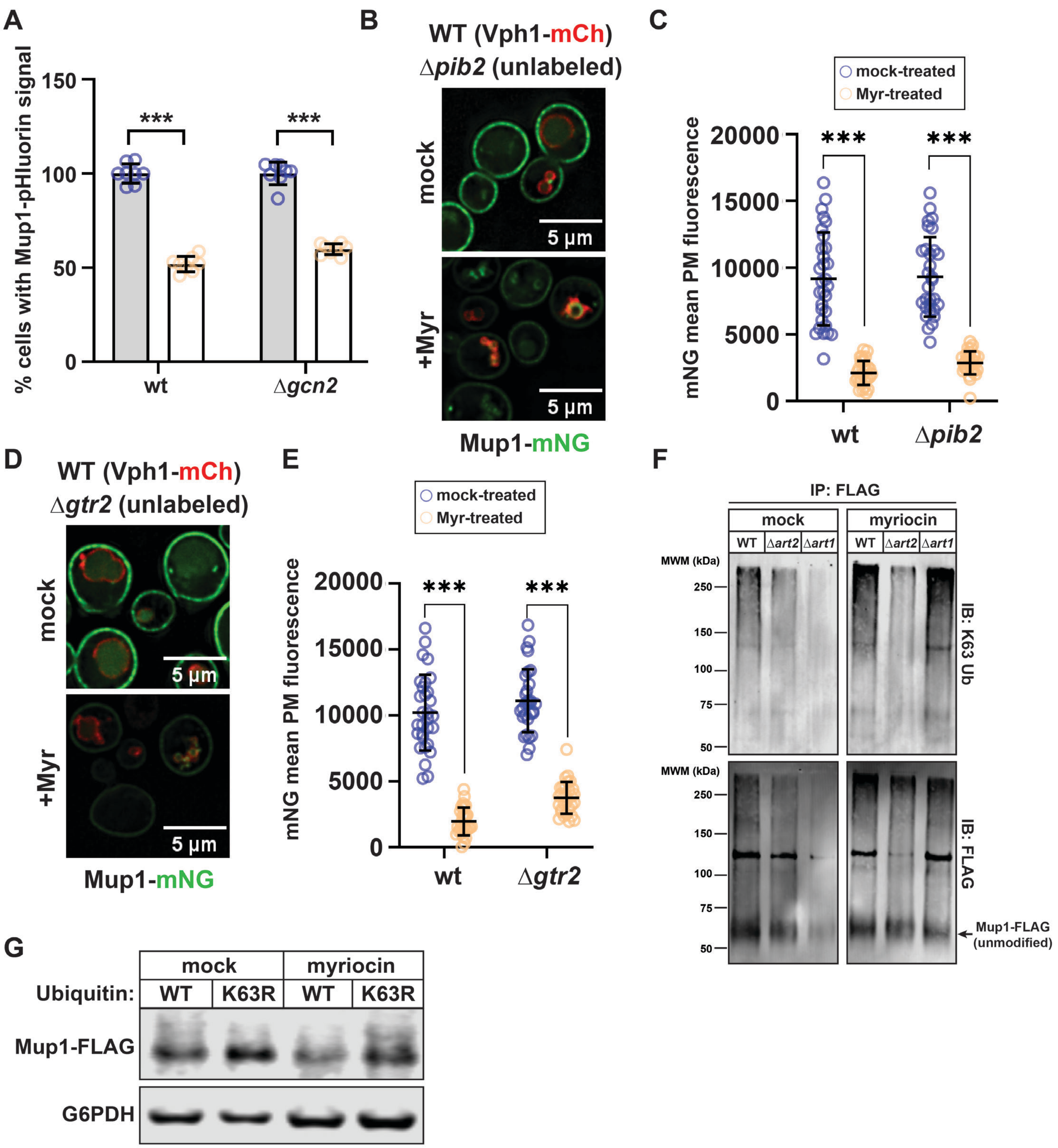

Journal of Cell Science • Supplementary information

Fig. S6. Analysis of factors required for Myr-triggered Mup1 endocytosis.

(A) Mup1-pHluorin fluorescence levels monitored following 5 hours of mock-treatment or Myr-treatment in yeast cells with the indicated genotypes (eight biological replicates; n = 10,000 cells for each biological replicate; ±SD (error bars)).

(B) Fluorescence microscopy images of cells expressing Mup1-mNG (green) following 5 hours of either mock-treatment or Myr-treatment. Cells expressing Vph1-mCherry (red vacuolar marker) are wildtype while cells with unlabeled vacuoles are *Δpib2* cells.

(C) Quantification of the results shown in FIG S6B. Mup1-mNG mean fluorescence at the PM of cells was measured after 5 hours of either mock-treatment or Myr-treatment (n = 30 cells).

(D) Fluorescence microscopy images of cells expressing Mup1-mNG (green) following 5 hours of either mock-treatment or Myr-treatment. Cells expressing Vph1-mCherry (red vacuolar marker) are wildtype while cells with unlabeled vacuoles are *Δgtr2* cells.

(E) Quantification of the results shown in FIG S6B. Mup1-mNG mean fluorescence at the PM of cells was measured after 5 hours of either mock-treatment or Myr-treatment (n = 30 cells).

(F) Mup1-FLAG was affinity purified from lysates from cells of the indicated genotype and analyzed by immunoblot using antibodies specific for K63 poly-ubiquitin and the FLAG epitope tag.

(G) Mup1-FLAG was analyzed by immunoblot of total cell lysates isolated from cells expressing either wildtype or K63R ubiquitin from a single source. G6PDH was used as a loading control.

Journal of Cell Science • Supplementary information

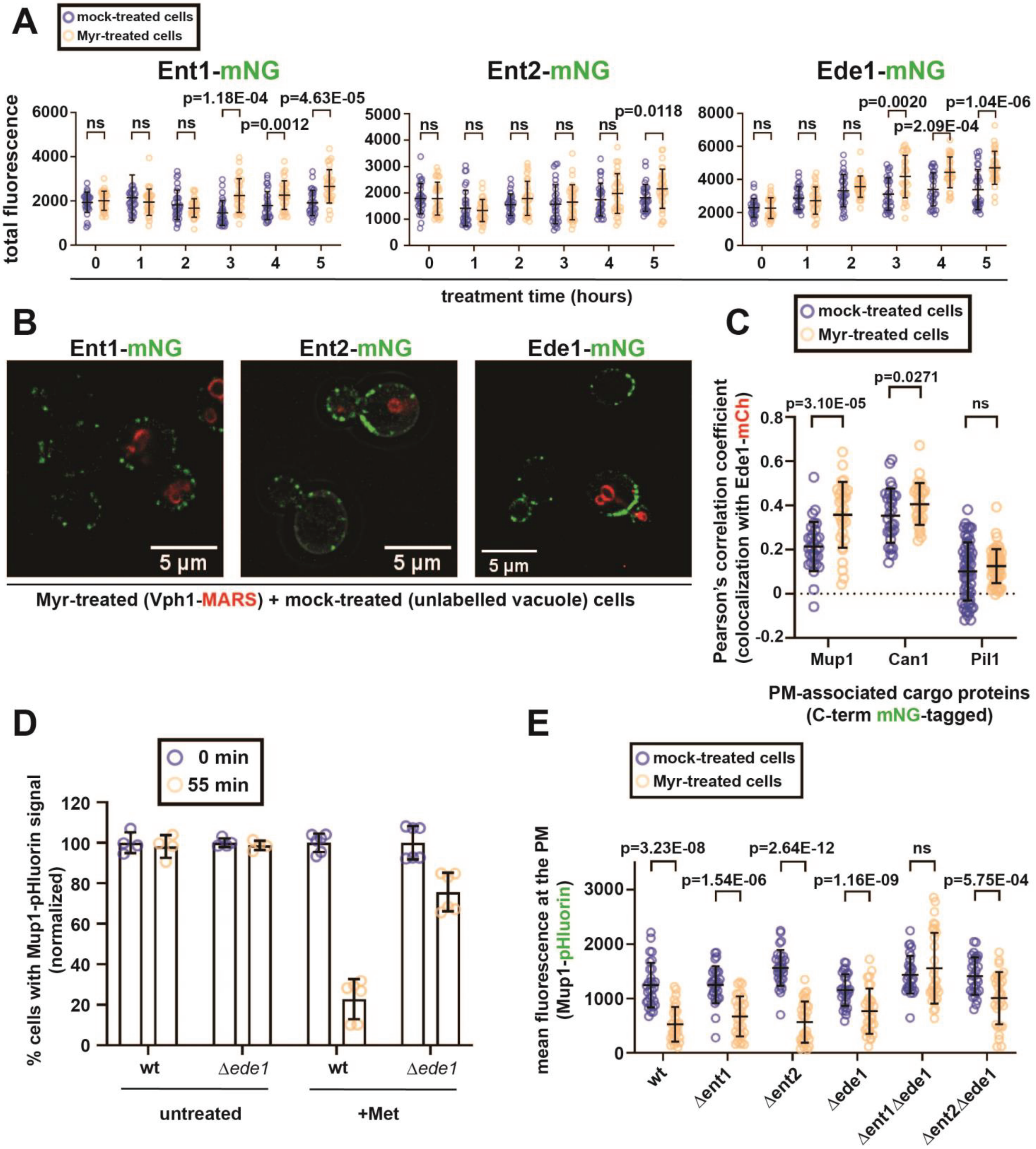

Journal of Cell Science • Supplementary information

Fig. S7. Analysis of the role of endocytic adaptors in Myr-triggered Mup1 endocytosis.

(A) Analysis of expression levels of different endocytic adaptor proteins (Ent1, Ent2, and Ede1) in mock-treated and Myr-treated cells. Yeast strains harboring endogenous C-terminal mNG tags for the indicated proteins were measured for total mNG fluorescence.

(B) Fluorescence microscopy images of cells expressing Ent1, Ent2, or Ede1 C-terminal fusions to mNG (green) following 5 hours of either mock-treatment or Myr-treatment. Cells expressing Vph1-MARS (red vacuolar marker) were Myr-treated while cells with unlabeled vacuoles were mock-treated.

(C) Fluorescence colocalization analysis was performed on cells expressing Ede1-mCherry and a selection of mNG-tagged PM integral proteins after 5 hours of mock-treatment or Myr-treatment.

(D) Mup1-pHluorin fluorescence levels in yeast cells with the indicated genotypes were measured before and after a 55 minute stimulation with methionine. Mup1-pHluorin signal was quantified using a flow cytometer (four or six biological replicates; n = 10,000 cells for each biological replicate; ±SD (error bars)).

(E) Cells expressing Mup1-pHluorin were visualized under a fluorescence microscope and the mean fluorescence of pHluorin at the PM was quantified using Fiji (n = 30 cells).

Journal of Cell Science • Supplementary information

Table S1. Strains used in this study.

Strain	Genotype	Reference
SEY6210	*MATα leu2-3,112 ura3-52 his3-Δ200 trp1-Δ901 suc2-Δ9 lys2-801*	Robinson et al. 1988
SEY6210.1	SEY6210 *MATa*	Babst et al. 1997
SUB280	*MATa lys2-801 leu2-3,112 ura3-52 his3-Δ200 trp1-1 [am] ubi1-Δ1::TRP1 ubi2-Δ2::ura3 ubi3-Δub-2 ubi4-Δ2::LEU2 (pUB39, pUB100)*	Finley et al. 1994
BY4741	*MATa his3-Δ1 leu2-Δ0 ura3-Δ0 met15-Δ0*	Brachmann et al. 1998
NHY999.1	SEY6210 *BAP2*::mNG::NATMX *VPH1*::mCherry::KanMX6	this study
BMY17.1	SEY6210 *BAP3*::mNG::NATMX *VPH1*::mCherry::KanMX6	this study
NHY423	BY4741 *CAN1*::mNG::NATMX *VPH1*::mCherry::KanMX6	this study
NHY492	SEY6210 *DIP5*::mNG::NATMX *VPH1*::MARS::*TRP1*	this study
BMY15	SEY6210 *GAP1*::mNG::NATMX *VPH1*::mCherry::KanMX6	this study
NHY988	SEY6210 *GNP1*::mNG::NATMX *VPH1*::mCherry::KanMX6	this study
NHY998.1	SEY6210 *HIP1*::mNG::NATMX *VPH1*::mCherry::KanMX6	this study
NHY496	SEY6210 *LYP1*::mNG::NATMX *VPH1*::MARS::*TRP1*	this study
NHY445	SEY6210 *MUP1*::mNG::NATMX *VPH1*::MARS::*TRP1*	this study
NHY944	SEY6210 *TAT2*::mNG::NATMX *VPH1*::mCherry::KanMX6	this study
NHY493	SEY6210 *HXT1*::mNG::NATMX *VPH1*::MARS::*TRP1*	this study
NHY494	SEY6210 *HXT2*::mNG::NATMX *VPH1*::MARS::*TRP1*	this study
NHY495	SEY6210 *HXT3*::mNG::NATMX *VPH1*::MARS::*TRP1*	this study
NHY503	SEY6210 *HXT6*::mNG::NATMX *VPH1*::MARS::*TRP1*	this study
NHY578	SEY6210 *ENT1*::mNG::NATMX *VPH1*::MARS::*TRP1*	this study
NHY579	SEY6210 *ENT2*::mNG::NATMX *VPH1*::MARS::*TRP1*	this study
NHY580	SEY6210 *EDE1*::mNG::NATMX *VPH1*::MARS::*TRP1*	this study
NHY674	BY4741 *PDR5*::mNG::NATMX *VPH1*::mCherry::*KanMX6*	this study
NHY1249.1	SEY6210 *MUP1*::mNG::NATMX *Δgtr2*::KanMX6	this study
NHY1241.1	SEY6210 *MUP1*::mNG::NATMX *Δpib2*::KanMX6	this study
JMY1811	SEY6210 *MUP1*::pHluorin::KanMX6	Lee et al. 2019
NHY807.1	JMY1811 *Δart1*::NATMX	this study
NHY814.1	JMY1811 *Δart2*::NATMX	this study
NHY858	JMY1811 *Δart3*::NATMX	this study
NHY860	JMY1811 *Δart4*::NATMX	this study
NHY862	JMY1811 *Δart5*::NATMX	this study
NHY864	JMY1811 *Δart6*::NATMX	this study
NHY866	JMY1811 *Δart8*::NATMX	this study
NHY868	JMY1811 *Δart9*::NATMX	this study
NHY909	JMY1811 *Δent1*::NATMX	this study
NHY913	JMY1811 *Δent2*::NATMX	this study
NHY917	JMY1811 *Δede1*::NATMX	this study
NHY1004.1	JMY1811 *Δent1*::NATMX *Δede1*::NATMX	this study
NHY928.1	JMY1811 *Δent2*::NATMX *Δede1*::NATMX	this study
NHY1006	JMY1811 *Δgcn2*::NATMX	this study
NHY413	*MUP1*::pHluorin::NATMX	Hepowit et al. 2021
NHY414	*MUP1*::pHluorin::NATMX *VPH1*::MARS::*TRP1*	Hepowit et al. 2021
NHY234.1	SUB280 *MUP1*::pHluorin::NATMX p*RS31Ub*	this study

NHY285	SUB280 *MUP1*::pHluorin::NATMX p*RS31Ub* K63R	this study
NHY746	SUB280 *MUP1*::mNG::NATMX p*RS31Ub*	this study
NHY744	SUB280 *MUP1*::mNG::NATMX p*RS31Ub* K63R	this study
NHY884	SUB280 *MUP1*::mNG::NATMX p*RS31Ub* *VPH1*::mRuby::KanMX6	this study
NHY688	SEY6210 *ENT1*::mNG::NATMX *MUP1*::mCherry::KanMX6	this study
NHY689	SEY6210 *ENT2*::mNG::NATMX *MUP1*::mCherry::KanMX6	this study
NHY687	SEY6210 *EDE1*::mNG::NATMX *MUP1*::mCherry::KanMX6	this study
NHY713	SEY6210 *MUP1*::mNG::NATMX *EDE1*::mCherry::KanMX6	this study
NHY719	SEY6210 *MUP1*::mNG::NATMX*EDE1ΔUBA*::mCherry::KanMX6	this study
NHY714	SEY6210 *CAN1*::mNG::NATMX *EDE1*::mCherry::KanMX6	this study
NHY734	SEY6210 *PIL1*::mNG::NATMX *EDE1*::mCherry::KanMX6	this study
NHY770	SEY6210 *ENT1*::mNG::NATMX *EDE1*::mCherry::KanMX6	this study
NHY716	SEY6210 *ENT2*::mNG::NATMX *EDE1*::mCherry::KanMX6	this study
NHY903	SEY6210 *ENT1*::mCherry::KanMX6 *MUP1*::pHluorin::NATMX	this study
NHY904	SEY6210 *ENT2*::mCherry::KanMX6 *MUP1*::pHluorin::NATMX	this study
NHY905	SEY6210 *EDE1*::mCherry::KanMX6 *MUP1*::pHluorin::NATMX	this study
NHY721	SEY6210 *EDE1*::mCherry::KanMX6 *CAN1*::mNG::NATMX	this study
NHY880	SEY6210 $MUP1_{1\text{-}566}$::mNG::NATMX	this study
NHY882	SEY6210 $MUP1_{1\text{-}571}$::mNG::NATMX	this study
JMY78	SEY6210 MUP1::3xFLAG::*TRP1*	Hepowit et al. 2021
JMY212	SEY6210 *Δart1*::HIS3 MUP1-3XFLAG::*TRP1*	this study
NHY870	SEY6210 *Δart2*::NATMX MUP1-3XFLAG::*TRP1*	this study
NHY1232	SEY6210 *MUP1*-3XFLAG::NATMX	this study
NHY1233	SEY6210 $MUP1_{1\text{-}551}$-3XFLAG::NATMX	this study
NHY1234	SEY6210 $MUP1_{1\text{-}566}$-3XFLAG::NATMX	this study
NHY1235	SEY6210 $MUP1_{1\text{-}571}$-3XFLAG::NATMX	this study
JTY240	SEY6210 *MUP1*::GFP::KanMX6 *VPH1*::MARS::*TRP1*	Tumolo et al. 2020
NHY1236.1	SUB280 p*RS31*-Ub wt MUP1::3xFLAG::NATMX	this study
NHY1237.1	SUB280 p*RS31*-Ub K63R MUP1::3xFLAG::NATMX	this study
NHY1243.1	SEY6210 *MUP1*::VN::KanMX6 *ART1*-VC::hph	this study
NHY1244.1	SEY6210 *MUP1*::VN::KanMX6 *ART2*-VC::hph	this study
NHY1052	SUB280 p*RS31*-Ub *MUP1*::VN::KanMX6 *EDE1*-VC::hph	this study
NHY1053	SUB280 p*RS31*-Ub K63R *MUP1*::VN::KanMX6 *EDE1*-VC::hph	this study

Journal of Cell Science • Supplementary information

Supplementary References

Babst, M., Sato, T. K., Banta, L. M. and Emr, S. D. (1997). Endosomal transport function in yeast requires a novel AAA-type ATPase, Vps4p. *EMBO J* **16**, 1820-31.

Brachmann, C. B., Davies, A., Cost, G. J., Caputo, E., Li, J., Hieter, P. and Boeke, J. D. (1998). Designer deletion strains derived from Saccharomyces cerevisiae S288C: a useful set of strains and plasmids for PCR-mediated gene disruption and other applications. *Yeast* **14**, 115-32.

Finley, D., Sadis, S., Monia, B. P., Boucher, P., Ecker, D. J., Crooke, S. T. and Chau, V. (1994). Inhibition of proteolysis and cell cycle progression in a multiubiquitination-deficient yeast mutant. *Mol Cell Biol* **14**, 5501-9.

Robinson, J. S., Klionsky, D. J., Banta, L. M. and Emr, S. D. (1988). Protein sorting in Saccharomyces cerevisiae: isolation of mutants defective in the delivery and processing of multiple vacuolar hydrolases. *Mol Cell Biol* **8**, 4936-48.
