# Supplementary material for: Art2 mediates selective endocytosis of methionine transporters during adaptation to sphingolipid depletion
Source: J Cell Sci. 2023 Jul 25;136(14):jcs260675. doi: 10.1242/jcs.260675 (PMC10399987; doi:10.1242/jcs.260675)
Supplement: Supplementary file 1 [file joces-136-260675_review_history.pdf]

Journal of Cell Science

Art2 mediates selective endocytosis of methionine transporters during adaptation to sphingolipid depletion

Nathaniel L. Hepowit, Bradley Moon, Adam C. Ebert, Robert C. Dickson and Jason A. MacGurn
DOI: 10.1242/jcs.260675

Editor: Daniel Billadeau

Review timeline

Original submission:	29 September 2022
Editorial decision:	8 November 2022
First revision received:	15 May 2023
Accepted:	1 June 2023

Original submission

First decision letter

MS ID#: JOCES/2022/260675

MS TITLE: Art2 mediates selective endocytosis of methionine transporters during adaptation to sphingolipid depletion

AUTHORS: Nathaniel L Hepowit, Bradley J Moon, Robert C Dickson, and Jason A MacGurn
ARTICLE TYPE: Research Article

We have now reached a decision on the above manuscript.

To see the reviewers' reports and a copy of this decision letter, please go to: https://submit-jcs.biologists.org and click on the 'Manuscripts with Decisions' queue in the Author Area. (Corresponding author only has access to reviews.)

As you will see, the reviewers raise a number of substantial criticisms that prevent me from accepting the paper at this stage. Most importantly, Reviewer 1 raises substantial concerns regarding the 'biology' being tested in the current manuscript, further characterization of the mechanism by which methionine levels are sensed and how Myr treatment affects this, and methods used in the manuscript for quantification of immunofluorescent images; also a concern of Reviewer 2. Reviewer 2 also requests quantitative immunoblotting for some key results, which I agree with. If you can address their concerns. If you think that you can deal satisfactorily with the criticisms on revision, I would be pleased to see a revised manuscript. We would then return it to the reviewers.

Please ensure that you clearly highlight all changes made in the revised manuscript. Please avoid using 'Tracked changes' in Word files as these are lost in PDF conversion.

I should be grateful if you would also provide a point-by-point response detailing how you have dealt with the points raised by the reviewers in the 'Response to Reviewers' box. Please attend to all of the reviewers' comments. If you do not agree with any of their criticisms or suggestions please explain clearly why this is so.

© 2023. Published by The Company of Biologists under the terms of the Creative Commons Attribution License (https://creativecommons.org/licenses/by/4.0/).

Reviewer 1

Advance summary and potential significance to field

The authors follow up a recently published study that documents that the drug myriocin (Myr), which impairs sphingolipid synthesis, increases lifespan in yeast
(PMID: 33744865). This previous study attributes differences in cellular pools of amino acids to the health of yeast cells (levels of 17 amino acids are depleted in these drug-treated cells) and notes that although a methionine transporter called Mup1 is synthesised and traffics normally to the surface, it is less active and has reduced overall surface levels.

This new work set out to test the hypothesis that other membrane transporters behave similarly, and collectively their reduced residency at the surface membrane explains the decreased cellular amino acid pools induced by Myr. The authors test a range of distinct surface transporter proteins but find none are downregulated like Mup1 in response to Myr. Over a dozen cargos are unaffected or have increased surface levels following addition of Myr, which inhibits bulk endocytosis measured by FM464, although how this block is imposed is not clear.

As no other cargos support the hypothesis, the latter part of the paper takes a mechanistic approach to understanding the unique response of Mup1 to Myr. The authors show the previously characterised (PMID: 32744498) Mup1 arrestin adaptor
(Art2) is responsible - via the E3 ligase Rsp5 - for endocytosis, in combination with the adaptors Ent1, Ede1 and ubiquitylation of Mup1.

In general, the experiments have been performed to a high level. For example, an impressive effort to capture distinct localisation phenotypes in the same microscopy experiment. However, the physiological relevance of the experiments is not well explained in the text and there are some experimental results that require further clarification - see specifics below.

Comments for the author

Major points
1. No tested explanation is provided as to why almost all the amino acid and glucose transporters increase surface residence or remain unchanged during the Myr time course where intracellular pools of amino acids and glucose are depleted.
Does this not argue that transporter trafficking is a bad metric to investigate increased longevity / decreased nutrient pools induced by Myr? Is it not more likely these cells are detecting amino acid starvation through a distinct mechanism and the increased surface levels of transporters are an indirect effect of cells desperately seeking to replenish nutrients?
2. A series of earlier papers (PMIDs: 30610170, 22118465, 18976803) have examined Mup1 endocytosis in response to specific stimuli, such as addition of substrate or nutrient limitation. These studies can be rationalised physiologically (eg avoid hyperaccumulation of substrate / promote degradation in response to nutrient limitation). It is not clear from this current study what process Mup1 endocytosis relates to following Myr treatment, especially as its behaviour is distinct from all other transporters tested. The idea this work is related to how “reduced sphingolipid biosynthesis promotes longevity” or that it provides insight to the “relationship between glucose metabolism and aging” is overstated in the text and the novelty and impact seem more constrained to characterising Mup1 trafficking in the presence of Myr without a biological context.
3. As Mup1 is the only protein that behaves as expected (degraded following Myr, which might account for reduced cellular pools of methionine) then the importance of methionine needs to be addressed. What senses methionine levels and why is Myr disrupting downstream responses to this? Furthermore, as the original study uses methionine auxotroph cells with deleted Mup1, this argues that other methionine uptake mechanisms are also involved and could be related to this response. Are Gap1 and Mup3 degraded in response to Myr? Are there genetic interactions between Mup1 and TORC1 regulator genes identified from the author’s previous study mentioned in abstract as (Gtr1/2 (mammalian Rags) and Vps34-Pib2?
4. Some relevant literature seems to have been omitted, which might help contextualise the current work. Relevant examples include: the study of surface transporters in presence of a synthetic derivative of myriocin (PMID: 29062000), the lipid mechanisms that affect surface

© 2023. Published by The Company of Biologists under the terms of the Creative Commons Attribution License (https://creativecommons.org/licenses/by/4.0/).

transporters following myriocin treatment (PMID: 25724885), the fact a single sphingolipid species can be recognised by transmembrane domains (PMID: 22230960).

Minor points
1. Previous work from the authors (PMID: 30610170; PMID: 27798240) has shown Mup1 tagged with GFP localises to the PM alone (with no Mup1 inside vacuole). In this work an mNeonGreen tagged version of Mup1 appears to significantly localise inside the vacuole (Fig. 1) under the same conditions. Is this new Mup1-mNG fusion protein trafficking to the vacuole more readily due to an issue (eg in folding), which is elevated in response to Myr? An explanation should be provided for this discrepancy. A comparison between GFP and mNG fusions to Mup1 could be performed. Previous experiments with pHluorin - that is not visible inside vacuole - or FLAG tag - that is degraded in the vacuole - are not suitable controls. Complementation experiments for Mup1 fusions would also provide better support for these constructs faithfully representing their untagged versions.
2. Colocalisation experiments of Mup1 and Art2 +/- Myr would make a better argument for this specific Mup1 response than the gene expression data shown in Supplemental Fig. 4. Particularly as Myr treatment appears to affect many arrestins - including those that act with surface transporters that are not being endocytosed in response to Myr.
3. The micrographs in Supplemental Fig 3 do not seem to match the Pearson's Correlation Coefficient values. Although the red signal appears more dotted at 30 min, I do not see any "yellow" overlapping signal. Based on this, one would expect the colocalisation values at 30 min to be more similar to 0 min, however these values are much closer to 60 min (in which the representative image shows almost all green/red signal overlapping). There are not sufficient details about how softWorx (ver. 7.0.0) was used to obtain these values and whether there is an error in setting thresholds.

Reviewer 2

Advance summary and potential significance to field

In this manuscript, Hepowit and colleagues characterize the effects on plasma membrane nutrient transporters during adaptation to decreased sphingolipid supply. Reports in multiple organisms indicate that inhibition of ceramide synthesis with myriocin (Myr) prolongs longevity. The authors here follow up on a previous observation that yeast treated wit myriocin exhibit reduced methionine uptake. The authors profile a latge number of plasma membrane transporters and find that many are increased at the plasma membrane upon Myr treatment which is consistent with their observation that bulk membrane endocytosis is also reduced. However, they find that the methionine transporter Mup1 is decreased at the plasma membrane due to selective increase in endocytosis upon Myr treatment. The authors mechanistically determine that enodcytosis in response to Myr does not require the machinery used to down-regulate Mup1 in response to excess methionine. Rather, nitrogen-starvation also down-regulates Mup1 and machinery from this pathway is used (Art2, Ede1, C-terminal ubiquitinylation K63). Unlike the nitrogen starvation response, Mup1 downregulation does not require Gcn2, and Ede1 and Ent1 function redundantly. These results raise interesting questions about how this selective endocytosis occurs in the face of decreased endocytic activity and shed light into a possible mechanism by which Myr promotes longevity. The manuscript is clearly written and the data justify the conclusions.

Comments for the author

Major comment:
1. The authors use C-terminal deletions to conclude that K567 but not K572 is required for Art2-dependent, Mup1 endocytosis. Since endocytosis is blocked by the D567-574 deletion, the authors can not rule out that the defect is due to other changes in the Mup1 mutant protein. Ubiquitinylation experiments should be repeated using single and double K567R and K572R mutants expressed either on a CEN plasmid or at endogenous locus.

Minor comments:
1. Fig 2A needs a legend to indicate which condition is Myr treated.
2. Y-axis labels for Supp Fig 4 are incorrect.

© 2023. Published by The Company of Biologists under the terms of the Creative Commons Attribution License (https://creativecommons.org/licenses/by/4.0/).

3. Line 255, "on" should read "no"

Reviewer 3

Advance summary and potential significance to field

In this study, Hepowit et al. made a surprisingg observation that myriocin treatment, which inhibits the sphingolipid synthesis, either has no effect or even stabilizes many plamsma membrane transporters in yeast. The only exception was Mup1, the PM methionine transporter. The authors then conducted a detailed study on the Myr-triggered Mup1 endocytosis and compared it to the methionine-triggered edosyctosis. They discovered that it requires Rsp5, Art2, and K63-dependent polyubiquitnation, which is different from the methinonne- triggered Mup1 endocytosis.
Overall, this is a solid study of the Myr effect on yeast plasma membrane proteins. The paper is well-written. The data quality is high. There is a robust statistical analysis on all data. I only have a few comments.

Comments for the author

1. The only major concern that I have is that the entire paper relies solely on the quantification of fluorescence images. Quanifying fluorence images is helpful but not as reliable as western blots. I realized that requesting the authors to repeat the whole paper with westerns would be unreasonable. But at least provide quantitative western blots for figures 4D-E (to show Art2 is required for Mup1 degradation), 5C-D (to show K567Δ; blocks Mup1 degradation), and 5G-H (to show K63R blocks Mup1 degradation). These results are the key findings of the paper and need to be confirmed by a second method. In addition, a detailed description of how the imaging quantification was done should be included in the text.
2. Fig S6A is not mentioned in the text.

First revision

Author response to reviewers' comments

Point-by-point Response to Reviewer Comments

[Reviewer comments in *blue*, author responses in **black**]

Reviewer 1 Comments for the Author:
Major points

1.No tested explanation is provided as to why almost all the amino acid and glucose transporters increase surface residence or remain unchanged during the Myr time course where intracellular pools of amino acids and glucose are depleted.
Does this not argue that transporter trafficking is a bad metric to investigate increased longevity / decreased nutrient pools induced by Myr? Is it not more likely these cells are detecting amino acid starvation through a distinct mechanism and the increased surface levels of transporters are an indirect effect of cells desperately seeking to replenish nutrients?

This is an excellent point. To address this, we mined data from a recently-published RNA seq analysis of the Myr-response in yeast (PMID: 36640272). This analysis suggests that a couple of the results we obtained (e.g., Hxt2 and Pdr5) do correspond to a transcriptional response. However, the rest of the changes in surface levels we report seem to occur independent of any transcriptional response. This new analysis is provided in **FIG S3**, and we added a paragraph to the results section (paragraph starting on **line 126**) to address this point.

© 2023. Published by The Company of Biologists under the terms of the Creative Commons Attribution License (https://creativecommons.org/licenses/by/4.0/).

2.A series of earlier papers (PMIDs: 30610170, 22118465, 18976803) have examined Mup1 endocytosis in response to specific stimuli, such as addition of substrate or nutrient limitation. These studies can be rationalised physiologically (eg avoid hyperaccumulation of substrate / promote degradation in response to nutrient limitation). It is not clear from this current study what process Mup1 endocytosis relates to following Myr treatment, especially as its behaviour is distinct from all other transporters tested. The idea this work is related to how "reduced sphingolipid biosynthesis promotes longevity" or that it provides insight to the "relationship between glucose metabolism and aging" is overstated in the text and the novelty and impact seem more constrained to characterising Mup1 trafficking in the presence of Myr without a biological context.

We agree with the reviewer that physiological context of this response is not as clear-cut as with other stimuli that have been studied. One recent study (PMID: 36640272) reported that blocking Mup1 endocytosis (by fusing to UL36) suppressed the longevity effects of Myr-treatment, providing evidence that Myr-mediated longevity depends on Mup1 endocytic clearance. Nevertheless, in our revised manuscript we have tried to avoid any overstatement or overinterpretation of our results.

3. As Mup1 is the only protein that behaves as expected (degraded following Myr, which might account for reduced cellular pools of methionine) then the importance of methionine needs to be addressed. What senses methionine levels and why is Myr disrupting downstream responses to this? Furthermore, as the original study uses methionine auxotroph cells with deleted Mup1, this argues that other methionine uptake mechanisms are also involved and could be related to this response. Are Gap1 and Mup3 degraded in response to Myr? Are there genetic interactions between Mup1 and TORC1 regulator genes identified from the author's previous study mentioned in abstract as (Gtr1/2 (mammalian Rags) and Vps34-Pib2?

We agree that the results of this study underscore the importance of methionine pools. In our revised manuscript, we included analysis of Myr-triggered Mup1 trafficking in *Δpib2* and *Δgrt2* mutant cells (**FIG S6B-E**) but we found these factors are not required. This result suggests that Mup1 clearance is upstream of any effects on TORC1 signaling. At the reviewer's request, we also looked at Gap1 and Mup3. Gap1 is constitutively sorted to vacuoles in the culture conditions and this is not altered by Myr treatment (**FIG S1A** and **Table 1**). Although we generated yeast with a C-terminal mNG tag on Mup3 we were never able to detect any fluorescent signal (data not shown) suggesting Mup3 isn't expressed under the culture conditions tested or the tagging disrupts expression.

4.Some relevant literature seems to have been omitted, which might help contextualise the current work. Relevant examples include: the study of surface transporters in presence of a synthetic derivative of myriocin (PMID: 29062000), the lipid mechanisms that affect surface transporters following myriocin treatment (PMID: 25724885), the fact a single sphingolipid species can be recognised by transmembrane domains (PMID: 22230960).

We are grateful to the reviewer for these suggestions – and we have incorporated each of this into the revised manuscript:

- PMID 29062000: see lines 215-216.
- PMID 25724885: see lines 133-134.
- PMID 22230960: see the new paragraph added to the Discussion section, beginning on line 346.

Minor points

1.Previous work from the authors (PMID: 30610170; PMID: 27798240) has shown Mup1 tagged with GFP localises to the PM alone (with no Mup1 inside vacuole). In this work an mNeonGreen tagged version of Mup1 appears to significantly localise inside the vacuole (Fig. 1) under the same conditions. Is this new Mup1-mNG fusion protein trafficking to the vacuole more readily due to an issue (eg in folding), which is elevated in response to Myr? An explanation should be provided for this discrepancy. A comparison between GFP and mNG fusions to Mup1 could be performed. Previous experiments with pHluorin - that is not visible inside vacuole - or FLAG tag - that is

© 2023. Published by The Company of Biologists under the terms of the Creative Commons Attribution License (https://creativecommons.org/licenses/by/4.0/).

degraded in the vacuole - are not suitable controls. Complementation experiments for Mup1 fusions would also provide better support for these constructs faithfully representing their untagged versions.

We have found that GFP signal is less stable inside the lumen of the vacuole compared to mNG, which is both brighter and more stable. This correlates with the pKa of the two proteins. For this study, mNG is a more sensitive reporter for vacuole trafficking, increasing our confidence in the evidence for cargo that do not exhibit increased trafficking in the context of Myr treatment. Additionally, we provide several orthogonal assays throughout the paper which provide multiple orthogonal lines of evidence to support Myr-triggered endocytic trafficking of Mup1.

2.Colocalisation experiments of Mup1 and Art2 +/- Myr would make a better argument for this specific Mup1 response than the gene expression data shown in Supplemental Fig. 4. Particularly as Myr treatment appears to affect many arrestins - including those that act with surface transporters that are not being endocytosed in response to Myr.

This is a great point, and we have included new experiments in the revised manuscript to address this. Specifically, we have added a split-fluorescence assay to monitor Mup1 proximity to adaptors in **FIG S5I-J**. The results reveal constitutive association between Art1 and Mup1, but a Myr-induced association between Mup1 and Art2.

3. The micrographs in Supplemental Fig 3 do not seem to match the Pearson's Correlation Coefficient values. Although the red signal appears more dotted at 30 min, I do not see any "yellow" overlapping signal. Based on this, one would expect the colocalisation values at 30 min to be more similar to 0 min, however these values are much closer to 60 min (in which the representative image shows almost all green/red signal overlapping). There are not sufficient details about how softWorx (ver. 7.0.0) was used to obtain these values and whether there is an error in setting thresholds.

This is an excellent point by the reviewer – and we apologize for this oversight. The original images shown were from a preliminary experiment that was not performed the same way and thus did not correspond directly to the quantification. In the revised manuscript we have included the images which directly correspond to the quantification (**FIG S4**). We have also included additional details in the methods regarding the quantification of this experiment.

Reviewer 2 Comments for the Author:
Major comment:
1. The authors use C-terminal deletions to conclude that K567 but not K572 is required for Art2-dependent, Mup1 endocytosis. Since endocytosis is blocked by the D567-574 deletion, the authors can not rule out that the defect is due to other changes in the Mup1 mutant protein. Ubiquitinylation experiments should be repeated using single and double K567R and K572R mutants expressed either on a CEN plasmid or at endogenous locus.

We appreciate the reviewer's comments and we agree with this suggestion. Originally, we focused on characterization of small C-terminal deletions of Mup1 because these can be generated chromosomally which minimizes cell-to-cell variation in Mup1 expression. Based on the reviewer's suggestion, we generated CEN plasmids expressing Mup1-GFP (WT, K567R, K572R, and the double point mutant) and we analyzed PM localization of these +/- Myr. The results are now included in **FIG 5D**. As expected with exogenous expression (even from a CEN plasmid) the cell-to-cell variation in these experiments is high. However, we still see a significant decrease in Mup1-GFP signal at the PM in response to Myr treatment, but not in the K567R mutant (or the double point mutant). These results are consistent with our C-terminal deletion results, and they reveal a requirement for K567 in the Myr-triggered endocytic clearance of Mup1.

Minor comments:
1. Fig 2A needs a legend to indicate which condition is Myr treated.

We apologize for this oversight, and we have added the legend to **FIG 2A** in the revised manuscript.

© 2023. Published by The Company of Biologists under the terms of the Creative Commons Attribution License (https://creativecommons.org/licenses/by/4.0/).

2. Y-axis labels for Supp Fig 4 are incorrect.

Again, we apologize for this oversight. The Y-axis labels for the revised figure (**FIG S5D**) have been corrected.

3. Line 255, "on" should read "no"

Thanks for catching this. It is corrected in the revised manuscript.

Reviewer 3 Comments for the Author:

1. The only major concern that I have is that the entire paper relies solely on the quantification of fluorescence images. Quanifying fluorence images is helpful but not as reliable as western blots. I realized that requesting the authors to repeat the whole paper with westerns would be unreasonable. But at least provide quantitative western blots for figures 4D-E (to show Art2 is required for Mup1 degradation), 5C-D (to show K567Δ blocks Mup1 degradation), and 5G-H (to show K63R blocks Mup1 degradation). These results are the key findings of the paper and need to be confirmed by a second method. In addition, a detailed description of how the imaging quantification was done should be included in the text.

To address this comment, we have augmented many of the figures by addition of new quantitative immunoblot analysis. This includes:

- Figure 4A: Myr-triggered clearance of Mup1
- Figure S5E-F: Effects of Myr on levels of ART expression
- FIG S5G-H: Art2-dependence clearance of Mup1 in response to Myr treatment
- Fig S6F: Art2-dependence of Myr-induced K63-liked ubiquitin polymers on Mup1
- Fig S6G: Myr-triggered clearance of Mup1 is dependent on K63 ubiquitin polymers

Additionally, we have expanded a description of our imaging quantification in the methods section of our paper.

2. Fig S6A is not mentioned in the text.

We apologize for this oversight. We have made sure all main figure and supplemental figure panels are called out in the revised manuscript.

<u>Second decision letter</u>

MS ID#: JOCES/2022/260675

MS TITLE: Art2 mediates selective endocytosis of methionine transporters during adaptation to sphingolipid depletion

AUTHORS: Nathaniel L Hepowit, Bradley J Moon, Adam C Ebert, Robert C Dickson, and Jason A MacGurn
ARTICLE TYPE: Research Article

I am happy to tell you that your manuscript has been accepted for publication in Journal of Cell Science, pending standard ethics checks.

© 2023. Published by The Company of Biologists under the terms of the Creative Commons Attribution License (https://creativecommons.org/licenses/by/4.0/).

Reviewer 1

Advance summary and potential significance to field

The summary I provided for the first submission is still relevant; most aspects discussed after are contextual.

Comments for the author

In this resubmission, the authors have added some nice data to this work, the split fluorescence data in both the main and supplemental figures is particularly nice and adds value to the story.
I do not fully understand why the transcriptomic analyses that has been added explains why the addition of Myriocin that triggers depletion of intracellular amino acids can be explained by only the down-regulation of a non-essential methionine permease. The discussion does a better job of matching the title, detailing this is a mechanistic dissection of a yeast protein following drug treatment that remodels sphingolipids. Future work will be important to understand how intracellular methionine levels and/or sphingolipid depletion might correlate with longevity at a mechanistic level.

Reviewer 2

Advance summary and potential significance to field

Revision

Comments for the author

The authors satisfactorily addressed comments from the initial review by examining the effects of individual lysine mutations rather than deletions in Mup1.

Reviewer 3

Advance summary and potential significance to field

The authors have adequately addressed all of my concerns.

Comments for the author

The authors have adequately addressed all of my concerns.

© 2023. Published by The Company of Biologists under the terms of the Creative Commons Attribution License (https://creativecommons.org/licenses/by/4.0/).
